# Methionine inducing carbohydrate esterase secretion of *Trichoderma harzianum* enhances the accessibility of substrate glycosidic bonds

**DOI:** 10.1186/s12934-024-02394-1

**Published:** 2024-04-26

**Authors:** Yang Liu, Tuo Li, Han Zhu, Linhua Cao, Lebin Liang, Dongyang Liu, Qirong Shen

**Affiliations:** 1 Key Lab of Organic-Based Fertilizers of China and Jiangsu Provincial Key Lab for Solid Organic Waste Utilization, Nanjing Agricultural University, Nanjing, 210095 People’s Republic of China; 2 College of Resource and Environmental Sciences, Nanjing Agricultural University, Nanjing, 210095 People’s Republic of China

**Keywords:** Polysaccharide hydrolysis, Carbohydrate esterase, Metabolomic, Xylan deacetylation, Glycosidic bond accessibility

## Abstract

**Background:**

The conversion of plant biomass into biochemicals is a promising way to alleviate energy shortage, which depends on efficient microbial saccharification and cellular metabolism. *Trichoderma* spp. have plentiful CAZymes systems that can utilize all-components of lignocellulose. Acetylation of polysaccharides causes nanostructure densification and hydrophobicity enhancement, which is an obstacle for glycoside hydrolases to hydrolyze glycosidic bonds. The improvement of deacetylation ability can effectively release the potential for polysaccharide degradation.

**Results:**

Ammonium sulfate addition facilitated the deacetylation of xylan by inducing the up-regulation of multiple carbohydrate esterases (CE3/CE4/CE15/CE16) of *Trichoderma harzianum*. Mainly, the pathway of ammonium-sulfate's cellular assimilates inducing up-regulation of the deacetylase gene (*Thce*3) was revealed. The intracellular metabolite changes were revealed through metabonomic analysis. Whole genome bisulfite sequencing identified a novel differentially methylated region (DMR) that existed in the *ThgsfR2* promoter, and the DMR was closely related to lignocellulolytic response. *Th*GsfR2 was identified as a negative regulatory factor of *Thce*3, and methylation in *ThgsfR2* promoter released the expression of *Thce*3. The up-regulation of CEs facilitated the substrate deacetylation.

**Conclusion:**

Ammonium sulfate increased the polysaccharide deacetylation capacity by inducing the up-regulation of multiple carbohydrate esterases of *T. harzianum*, which removed the spatial barrier of the glycosidic bond and improved hydrophilicity, and ultimately increased the accessibility of glycosidic bond to glycoside hydrolases.

**Supplementary Information:**

The online version contains supplementary material available at 10.1186/s12934-024-02394-1.

## Background

Plant biomass is a large-scale renewable organic carbon resource, and the application of microorganisms to degrade and convert it into biofuels and biochemicals is a promising way to solve the current energy crisis [1]. Plant fiber components are complex and mechanically dense, mainly composed of cellulose (40%–50%), hemicellulose (20%–40%), and lignin (20%–30%) [2]. Its whole-component degradation requires the combination of several lignocellulolytic carbohydrate active enzymes (CAZymes) [3]. The CAZymes family consists of Glycoside Hydrolases (GHs), Glycosyl Transferases (GTs), Polysaccharide Lyases (PLs), Carbohydrate Esterases (CEs), and Auxiliary Activities enzymes (AAs), which are secreted by saprophytic microorganisms to act on lignocellulose and ultimately hydrolyze glycosidic bonds to form oligosaccharides. Trichoderma species are typical saprophytic fungi [4] that generally possess an affluent CAZymes system and a complete pentose (C5) and hexose (C6) utilization pathway. Meanwhile, it can regulate CAZymes secretion and hydrolysis strategy based on different lignocellulosic substrates. The major lignocellulosic response regulators are the transcriptional activator ACE2, transcriptional repressor ACE1 [5], zinc-finger transcription factor PACC, CCAAT binding complex HAP2/3/5, glucose repressor CRE1, and the GATA factor AREA, which form a regulatory network controlling the expression of lignocellulases [6, 7].

Substrate variability has a significant impact on the degradation efficiency of CAZymes. Lignin degradation is relatively complicated, it is mainly an amorphous non-homogeneous phenolic polymer composed of coumarin, coniferyl alcohol, and sinapyl alcohol [8], mainly degraded by laccase and lignin peroxidase [9]; Cellulose is the most critical component of plant cell wall and structural support material, which is also regarded as the linear polysaccharide composed of glucose linked through glycosidic bonds. GHs such as Endoglucanases (EG), cellobiohydrolase (CBH), and β-glucosidases (BG) act directly on glycosidic bonds, and their catalytic function generally comes from two amino acid residues: proton donor and nucleophile/base [10]. Hemicellulose is mainly composed of xylose, arabinose, and mannose polymerization. Xylanase, mannanase, and arabinase are the main hemicellulases [11, 12]. Hemicellulose also has diverse chemical modifications, such as methylation, acetylation, and ferulic acylation, which greatly limits the hydrolysis efficiency of hemicellulase and even other lignocellulosic components, especially the acetylation of xylose residues, which decreases the hydrophilicity of fibers [13, 14] and even affect the binding efficiency between cellulase and cellulose [15]. Acetylation of hemicellulose is prevalent in gramineae, which facilitates the interaction between polysaccharides [16], thus improving the resistance to physical and biological stresses [17, 18]. Nevertheless, the acetylation remaining in plant residues will greatly limit the degradation of hemicellulases, which is a bottleneck for plant biomass saccharification.

Trichoderma species can secrete carbohydrate esterases (CEs) such as CE16, CE5, and CE3, which can catalyze the deacetylation of polysaccharides [19]. The CAZymes Database (CAZyDB) shows that the family number of CEs is only 20, whereas the GHs have the most expansive gene family in CAZymes (GH family number: 184). Although this result is not statistical for a single species, it implies that the GHs family is abundant in microorganisms. CEs are unappreciated because of the more contracted gene family compared to GHs and cannot act directly on glycosidic bonds. However, its polysaccharide deacetylation function can remove the obstacles to the hydrolysis of glycosidic bonds by GHs. The improvement of GHs hydrolysis efficiency means that cells can obtain sufficient carbon sources with less enzyme production, while the saved energy will be used for secondary metabolism and hyphae growth. Using Trichoderma species as a cell factory to achieve industrial bioconversion of straw into chemicals requires the support of huge hyphae biomass. Owing to the nutrition of straw being relatively single, a small amount of amino acids or ammonium sulfate are often added during solid fermentation [20], which can significantly stimulate the straw utilization efficiency of filamentous fungi. However, the nitrogen/sulfur supplied from these supplements is insignificant for supporting long-term microbial life activities, so it is more likely that the cellular assimilation products of added ammonium sulfate act as triggers to stimulate the lignocellulolytic response.

Here, we report that methionine, the major metabolite of ammonium sulfate, can enhance the lignocellulose degradation efficiency of *Trichoderma harzianum* by promoting its polysaccharide deacetylation capacity. *T. harzianum* is a plant residue degrader [21–23] with a rhizosphere-promoting function [24, 25], which is capable of degrading soil polysaccharides that cannot be utilized by plants and converting them into phytohormone such as IAA through cellular metabolism [26]. Therefore, *T. harzianum* can not only serve as a cell factory for plant biomass to biochemicals conversion but also for phytohormone synthesis [4], all of which rely on more efficient polysaccharide degradation ability. This study reveals a connection between ammonium sulfate assimilation and microbial lignocellulose degradation strategies, which helps to further explore the potential of Trichoderma species in plant biomass bioconversion.

## Materials and methods

### Strains and plasmids

Plasmids were propagated in *Escherichia coli* DH5α (Tsingke Biotechnology, China). DH5α were cultured in LB medium (tryptone, 10 g L^−1^; yeast extract, 5.0 g L^−1^; NaCl, 5.0 g L^−1^). The *Trichoderma harzianum* strain used in this study was named *T. guizhouense* NJAU4742 [27] (Genomic NCBI ID: LVVK00000000.1, Genetic database: https://bioinfo.njau.edu.cn/tgn4742/index.php). The WT and mutants were cultured on PDA/PDB (Oxoid, UK) or MM (Carbon source, 10 g L^−1^; KH_2_PO_4_, 15 g L^−1^; (NH_4_)_2_SO_4_, 10 g L^−1^; MgSO_4_, 0.6 g L^−1^; FeSO_4_, 5.0 mg L^−1^; MnSO_4_, 1.6 mg L^−1^; ZnSO_4_, 1.4 mg L^−1^; CoCl_2_, 2.0 mg L^−1^; CaCl_2_ 1.0 g L^−1^) at 28 °C. T1, T2, and T3 were prepared by using different AS content liquid MM to obtain the 75% water-content straw. The plasmid pAbAi, pGADT7, and Matchmaker Gold Yeast (Takara, Japan) were used in the Y1H assay. Positive Y1H transformants were screened with SD-Ura-Leu medium and Protein-DNA interactions were verified with SD-Ura-Leu + AbA.

### Enzyme activity assay

The extracellular proteins (CAZymes) were extracted by 50 mL NaAc buffer (NaAc, 100 mM; HAc, 10 mM). The EG/Xylanase/FPase activity assay was performed as follows: 480 μL CMCNa (0.5%, W/V)/480 μL Xylan (0.5%, W/V)/Whatman filter paper, 500 μL NaAc buffer, and 20 μL crude enzyme solution were mixed and incubated at 50 ℃ for 20 min. Then 1 mL DNS (NaKC_4_H_4_O_6_, 185 g L^−1^; NaOH, 20.1 g L^−1^; C_7_H_4_N_2_O_7_, 6.3 g L^−1^; Na_2_SO_3_, 5 g L^−1^; C_6_H_5_OH, 5 g L^−1^) was added and heated by boiling water for 10 min, after which the absorbance of different treatments were determined at OD_520_. OD was converted to enzyme activity by standard curve, per enzyme activity unit was defined as the amount of enzyme required to liberate 1 µmol glucose per minute.

### Biomass assay

The hyphae and medium (straw) were fully mixed, and 10.0 g sample was accurately weighed. The samples were rapidly frozen with liquid nitrogen and milled. DNA of hyphae was extracted by PowerSoil Pro Kit (QIAGEN, Germany) according to the manufacturer's instruction and then dissolved with 20 μL ddH_2_O. The copies of standard DNA solution was 4.5 × 10^10^ copy μL^−1^ (2000 bp, 100 ng μL^−1^), which was diluted 10 folds successively. The standard regression equation was Y = (34.3 − X)/3.2, R^2^ = 0.999, Y = log_10_^(template copies)^, X = average Cts. The Cts of treatments and mutants were determined by qPCR, and Cts were converted to genome copies by the standard regression equation.

### Gene editing assay

The homologous arms (HA) were amplified through PCR. The hygromycin phosphotransferase gene (*hph*) was used as the biomarker. The structure of functional fragments for knockout and overexpression was “HA1–*hph*–HA2” and “HA–*hph*–strong promoter–genebody”, respectively. Spores were cultured on PDA at 28 ℃ for 16 h, the newly germinated spores were lysed by fungal cell-wall lyase (SIGMA, US), and the protoplasts were collected. The PEG/CaCl_2_ method was used for functional fragment transformation. Protoplasts, 400 μL Solution B (CaCl_2_·2H_2_O, 0.5 M; Sorbitol, 10 M; 5 mM pH 7.5 Tris–HCl), 100 μL PEG solution (CaCl_2_·2H_2_O, 0.5 M; PEG6000, 25%, W/V; 5 mM pH 7.5 Tris–HCl), and 20 μL DNA (200 ng μL^−1^) were mixed gently, and 2 mL PEG solution was added after 20 min incubation. Finally, 3 mL Solution B was added, and the mixture was uniformly coated on sucrose PDA (PDA, 39 g L^−1^; sucrose, 1 M), and then covered with hygromycin PDA (Hygromycin B, 0.2 g L^−1^) after cultured at 28 ℃ for 18 h [28]. The transformants were verified by PCR and the correct transformants should be verified again after passage.

### Metabolomics analysis

The hyphae of different treatments were frozen by liquid nitrogen and then fully ground, followed by extraction with sterile distilled water. The extracts were identified using a quadrupole orbitrap mass spectrometer based on the LC–MS/MS system, and 40,850 peaks were obtained, while 26,383 peaks were retained after filtering for deviations and missing values [29]. The data were logarithmically transformed and centered, and then analyzed by automatic modeling. Subsequently, UV formatting and OPLS-DA modeling analysis were performed for the principal component. The quality and validity of the model were judged by R^2^X, R^2^Y, and Q^2^Y were obtained after cross-validation. R^2^X and R^2^Y denoted the interpretability of the OPLS-DA model on the information of X and Y matrices, respectively, and Q^2^Y was used to evaluate the predictiveness [30]. The adjusted data were compared with database and combined with the qualitative and quantitative results for univariate statistical analysis (UVA) and multivariate statistical analysis (MVA). Subsequently, the metabolites with significant differences were screened by ROC analysis, and the AUC value closer to 1 was considered to have a better diagnostic effect, concurrently, the AUC value higher than 0.9 indicated a high accuracy [31]. Finally, a series of bioinformatics analyses were performed to visualize the biological functions of differential metabolites.

### WGBS assay

2 µg hyphae DNA extracted from different treatments was diluted to 10 µL, and 1.1 µL NaOH (3 M) was added, after which the system was incubated at 37 °C for 10 min; then 6 µL hydroquinone (10 mM), 104 µL sodium bisulfite (3.6 M) was added, and sealed with mineral oil, respectively, and then the system was incubated at 50 °C for 18 h. Subsequently, the DNA sample was recovered, and the bisulfite conversion was validated by methylation-specific PCR (MSP). The pretreated DNA was ultrasonically fragmented to 200–300 bp and used to construct the DNA library. The libraries were sequenced by using the Illumina sequencing platform. The Fastp software was used to obtain clean reads [32], and Bismark [33] software with bisulfite conversion algorithm was used to correctly locate the reads and count the conversion rate after being treated with bisulfite. MethylKit [34] software was used to complete the detection of methylation sites and count the methylation at genome-wide and gene element levels. DMRs analysis was also performed by MethylKit, and the methylation level data within genome tiling windows were obtained. The logistic regression model was used to analyze the DMRs between groups and then analyzed the methylation level differences. Eventually, the DMRs were annotated according to the information of protein-coding genes.

### ChIP-seq assay

His-tag was added to the C-terminus of *Th*GsfR2 and verified by sequence and western blot. Hyphae of the modified strain were immersed in cross-linking buffer (EDTA, 1.0 mM; FMSF, 1 mM; Formaldehyde, 1%, V/V, pH 8.0 Tris–HCl, 10 mM;) for 25 min, and terminated by 10 × Glycine (Beyotime, China). The chromatin was ultrasonically broken to 200–1000 bp. 200 μL supernatant was obtained by centrifuge, 1.8 mL ChIP diffusion buffer (Beyotime, China) and 1 μg 6 × His-tag ChIP-class antibody (Abcam, UK) was added, and incubated at 4 ℃ overnight. Then, 60 μL Protein A/G beads were added into the ChIP system and incubated at 4 ℃ for 5 h. The supernatant was removed by centrifuge and the sediment was cleaned with Low salt immune complex wash buffer, High salt wash buffer, LiCl wash buffer, and TE buffer in turn. Finally, 500 μL elution buffer (NaHCO_3_, 0.1 M; SDS, 1%, W/V) was added to elute the Protein-DNA complex and the nucleic acid was recovered after de-crosslinking (5 M NaCl).

NGS: The recovered DNA was subjected to high-throughput sequencing (Illumina HiSeqTM2000) after library construction to obtain raw sequenced reads. The clean data was then aligned to the reference genome using BWA (version: 0.7.15) [35] and the bam files were obtained, the duplicate sequences were removed and the only aligned sequences were retained; the peak information was analyzed genome-widely using MACS (version:2.1.1) [36], and the screening threshold for the significant peak was q-value < 0.05. Peak detection was performed to obtain information of enriched regions, and the distribution of peak, nearest gene search and motif prediction were performed. Finally, the peak distribution was counted, and GO, KEGG enrichment and transcription factor prediction were performed for nearest peak genes.

### Yeast one-hybrid assay

The conserved sequence “TCTCTCTCTCT” of motif1 was inserted into the multiple cloning sites (MCS) of pAbAi, and the *Th*GsfR2 was expressed by pGADT7. The transformation was carried out based on the PFG/LiAc method, 100 μL Gold Yeast, 50 μL NaCl (0.9%, W/V), 5 μL DTT (1 M), 500 μL PEG Mix (45 mL 50% PEG3350; 5 mL 1 M LiAc; 500 μL 1 M Tris–HCl, 100 μL 0.5 M EDTA) and 1 μg BstBI linearized pAbAi::motif1 were mixed and incubated at 30 ℃, and 20 μL DMSO was added, the Yeast was heat shocked at 42 °C for 15 min, and then incubated on ice for 5 min [37]. After resuscitation, transformation system was coated on SD-Ura medium and cultured at 30 ℃ for 3 days. 10 μL positive transformant (Bait-reporter) was dropped onto SD-Ura + AbA (600 ng mL^−1^) for self-activation verification. The pGADT7::*Th*GsfR2 was transformed into the Bait-reporter, and 10 μL positive transformant was dropped onto SD-Ura-Leu + AbA (600 ng mL^−1^) and then cultured at 30 °C for 3 days, until the colony formed.

### HSQC assay

To remove the pectin and lignin, 1.0 g rice straw without hyphae was incubated in 5 mL ammonium oxalate (1%, w/v) at 37 °C, and then the pellets were incubated in 3 mL 11% peracetic acid at 80 °C for 40 min. Xylan was extracted by DMSO at 70 °C and pelleted by 5 volumes of ethanol/methanol/water (7/2/1, pH 3.0). 10 mg extracted xylan was dissolved in 0.5 mL deuterated DMSO-d_6_ (Sigma-Aldrich, USA) and subjected to ^1^H–^13^C HSQC [38, 39]. HSQC spectrum obtained by Bruker 600-MHz NMR spectrometer. The standard pulse sequence (gHSQCAD) was used to determine the one-bond ^13^C–^1^H correlation. The HSQC spectrums were collected with a spectrum width of 10 ppm in ^1^H dimension and 200 ppm in ^13^C dimension. The spectrums were calibrated by the DMSO solvent peak (dC 39.5 ppm, dH 2.49 ppm) [40]. The acetyl-group identification in NMR data and peak area statistics were conducted with MestReNova (Version 10.0.2) software.

### WSI determination

After removing hyphae, the rice straw from each treatment was dried to a constant weight, and 1.000 g samples were weighed under an infrared lamp and placed in a sample holder, subsequently transferred to a designated position inside a magnetic suspension balance. The samples were degassed at 120 °C for 3 h under vacuum conditions and then cooled to 28 °C [41]. The evaporator was then connected to the adsorption room and the mass of adsorbed water was recorded after reaching the saturation pressure. The process of vacuum drying and degassing was subsequently repeated to obtain the water adsorption isotherm by setting the vapor pressure gradient.

### Low-field NMR

WT was inoculated into the rice straw medium including T1 and T3 at 28 °C for 15 days. After removing hyphae, the straw was dried to constant weight and ground through a 100-mesh sieve. 1.000 g straw powder from T1 and T3 were weighed, and 1 mL deionized water was added and mixed well, then stood overnight. The mixtures were centrifuged at full speed for 10 min and the precipitates were subjected to the *T*_2_ relaxation time determination. The NMR analyzer (MesoMR23-060H-I, Niumag) operating frequency was 18 MHz, and the operating temperature was 32 °C, while the pulse sequence was Carr-Purcell-Meiboom-Gill (CPMG) [42]. The repeat sampling waiting time was 3000 ms, echo time was 0.1 ms, echo number was 1000, and radio frequency delay was 0.08 ms. Data was collected to generate *T*_2_ relaxation spectra using Origin 2023b.

## Results and discussion

### Ammonium-sulfate facilitated plant biomass utilization of *T. harzianum*

**Fig. 1:**
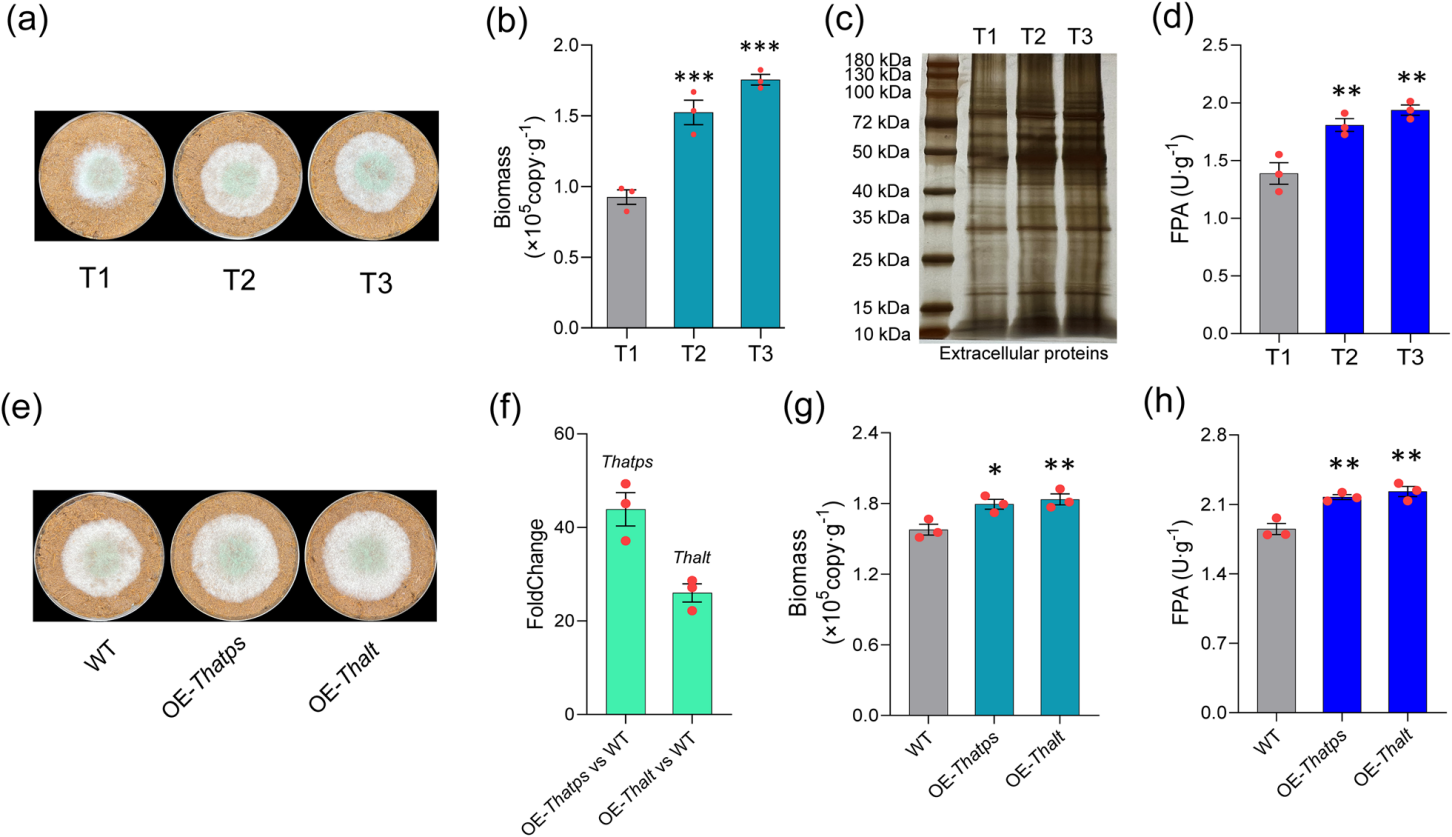
Ammonium sulfate significantly improved the lignocellulose degradation of *T. harzianum*. **a** Growth of WT in rice straw medium with different ammonium sulfur addition (T1: 0%, T2: 0.5%, T3: 1.0%); **b** The hyphae biomass of T1, T2, and T3. Notably, the biomass of hyphae growing on straw was determined by absolute qPCR. **c** Extracellular proteins from each treatment (T1, T2, T3) were solubilized by using 10 mL PBS buffer, and 40 μL were subjected to SDS-PAGE and silver-stained. **d** The FPA of T1, T2, and T3. **e** The growth of WT, OE-*Thatps*, and OE-*Thalt* on MM + straw, and the strains were cultured at 28 ℃ for 4 days. **f** Relative to WT, and *Thatps* was 44-folds up-regulated in OE-*Thatps*, *Thalt* was 44-folds up-regulated in OE-*Thalt*. **g** Biomass of WT, OE-*Thatps*, and OE-*Thalt*. **h** FPA of WT, OE-*Thatps*, and OE-*Thalt*. Bars represented mean ± SEM, with n = 3 biological repeats; red dots resembled values from individual experiments. Student’s *t*-testing was conducted in (**b**, **d**, **g**, **h**). ***significant difference to WT at two-tailed *P* = 0.000 (b, T2), 0.000 (**b**, T3); **significant difference to WT at two-tailed *P* = 0.005 (**d**, T2), 0.001 (**d**, T3), 0.007 (**g**, OE-*Thalt*), 0.003 (**h**, OE-*Thatps*), 0.001 (**h**, OE-*Thalt*); *significant difference to WT at two-tailed *P* = 0.014 (**g**, OE-*Thatps*)

Different ammonium-sulfate (AS) gradients including T1 (0%), T2 (0.5%), and T3 (1%) were set by adjusting the mineral medium with rice straw as carbon source (MM + straw), and other mineral nutrients content were kept consistent. The wild-type (WT) of *T. harzianum* was inoculated on the treatments for 4 days (Fig. 1a). Since hyphae could not be isolated well from the straw medium, we determined biomass by using absolute quantitative PCR, and the result showed that the hyphae biomass of T2 (1.52 × 10^5^ copy·g^−1^) and T3 (1.76 × 10^5^ copy·g^−1^) were significantly greater than T1(0.93 × 10^5^ copy·g^−1^), indicating that the biomass was increased gradually with the AS gradient (Fig. 1b). Extracellular proteins from each treatment were extracted by the same volume of PBS buffer (10 mL) and subjected to SDS-PAGE (Fig. 1c), which showed that the extracellular proteins contents were increased with the AS gradient (Additional file 1: Fig. S1a, b). In addition, the FPA, EG, and xylanase activities of T2 and T3 were also significantly increased than that of T1 (Fig. 1d; Additional file 1: Fig. S6a). All these results indicated that small amounts of ammonium-sulfate could significantly induce an increase in the lignocellulose utilization capacity of *T. harzianum*. In order to exclude the physical interference of exogenous addition, we respectively overexpressed the key enzymes in ammonium-sulfate assimilation pathway, ATP sulfatase (*Thatps*) and alanine transaminase (*Thalt*, KEGG ID: K00814, GENE ID: A1A111846.1, NCBI ID: OPB36402.1), which catalyzes sulfate reduction and ammonia transfer to pyruvate and obtained the strains OE-*Thatps* ( *Thatps* was 44-folds up-regulated) and OE-*Thalt* (*Thalt* was 26-folds up-regulated) (Fig. 1f). The mutants and WT were cultured on MM + straw (with same AS content) for 4 days (Fig. 1e). The biomass and CAZymes activities (FPA, EG, and Xylanase) of OE-*Thatps* and OE-*Thalt* were also significantly greater than that of WT (Fig. 1g, h; Additional file 1: Fig. S6b), suggesting that the enhanced AS assimilation capacity was conducive to the lignocellulolytic utilization of *T. harzianum*, and also hinting at a potential linkage between AS assimilation pathway and lignocellulolytic response.

### Ammonium sulfate induced a significant increase in intracellular methionine and 5-methylcytosine

**Fig. 2:**
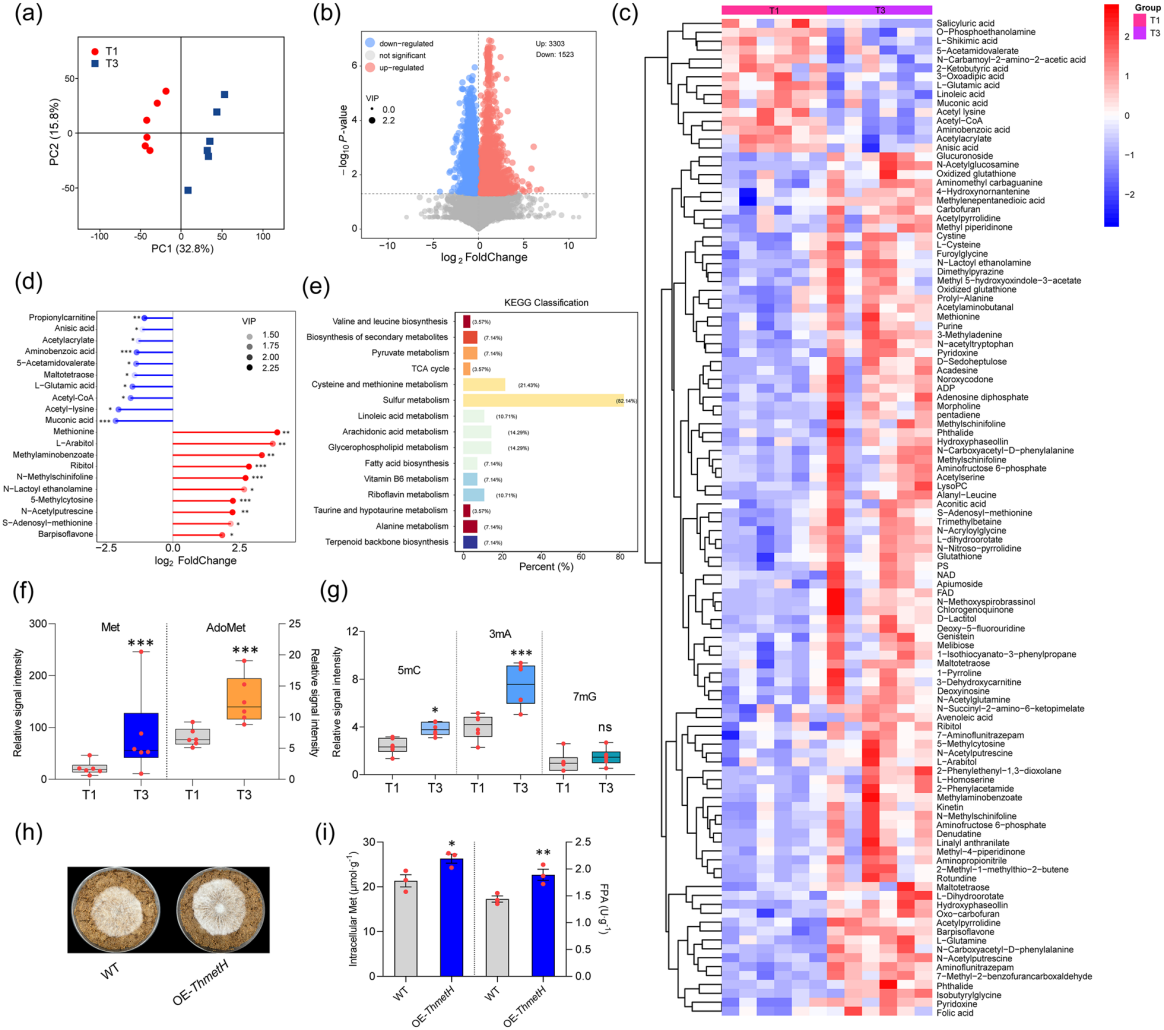
Metabolomic revealed the changes in intracellular metabolites induced by ammonium sulfate. **a** PCA demonstrated intra-group reproducibility and inter-group variability for T1 and T3. **b** The volcano plot counted and displayed log_2_FoldChange of the differential metabolites between T3 and T1 and log_10_*P*-value, with each point representing a differential metabolite. **c** Heatmap showed the significantly (*P* < 0.05) up/down-regulated differential metabolites identified by secondary mass spectrometry. **d** Match plot showed the significantly up/regulated differential metabolites (VIP-value > 1.5). **e** KEGG enrichment of differential metabolic pathways. **f** MS signal intensity of Met and AdoMet in T1 and T3. Notably these values were all significantly greater in T3 than T1**. g** MS signal intensity of 5mC, 3mA, and 7mG in T1 and T3. **h** Growth comparison of OE-*ThmetH* and WT on MM + straw. **i** Quantification of intracellular Met content and FPA. Intracellular Met content and FPA of OE-*ThmetH* were significantly higher than that of WT. All results were obtained from hyphae samples grown on T1 and T3, both of which contained 6 biological replicates; red dots resemble values from individual experiments. Student’s *t*-testing was conducted in (**f**, **g**, **i**), *significant difference to T1 at two-tailed *P* = 0.022 (g, T3: 5mC), *significant difference to WT at two-tailed *P* = 0.011 (i, OE-*ThmetH*: intracellular Met). **significant difference to WT at two-tailed *P* = 0.004 (**i**, OE-*ThmetH*: FPA); ***significant difference to T1 at two-tailed *P* = 0.000, (**f**, T3: Met), 0.000 (**f**, T3: AdoMet), 0.000 (**g**, T3: 3mA); ns = no statistical difference to T1 at two-tailed *P* = 0.454 (**g**, T3: 7mG)

AS needs to be assimilated by cells to generate relevant metabolites to perform its regulatory function, therefore, we performed the metabolomic analysis on T1 and T3 to reveal the changes in intracellular metabolites. PCA exhibited a significant difference between T1 and T3, and good reproducibility of 6 biological repeats (Fig. 2a). The correlation between metabolites and sample categories was modeled by OPLS-DA. The model validity was judged by using cross-validation, and result indicated that the model with high interpretability for categorical variables (R^2^Y = 0.995, *P* < 0.05) and high predictability (Q^2^ = 0.807, *P* < 0.05) (Additional file 1: Fig. S2a). The volcano plot showed the metabolites content Foldchange in T3 relative to T1, with 3303 metabolites up-regulated and 1523 metabolites down-regulated in T3 (Fig. 2d). Signal intensity changes of metabolites were identified by mass spectra (LC–MS/MS) by comparing the 6 biological repeats of T3 and T1, and the metabolites from database comparison (score > 0.9) were then subjected to clustering analysis. The results showed that amino acids, especially for sulfur-containing amino acids, were the major up-regulated metabolites, while organic acid metabolites were the major down-regulated substances. Notably, a large number of methylation-modified metabolites were upregulated (Fig. 2c). Matchstick plot showed the 10 most significantly up/down-regulated metabolites in volcano plot, and statistical significance analysis was performed. Methionine was the most significantly up-regulated metabolite, while muconic acid was the most significantly down-regulated (Fig. 2d). KEGG enrichment showed that mainly various amino acid metabolic pathways responded to AS addition, among which sulfur metabolism and methionine synthesis pathway were the most drastically affected by AS addition (Fig. 2e).

To screen out the signature metabolite that can serve as a marker capable of distinguishing different AS additions (T1 and T3), the receiver operating characteristic (ROC) analysis was performed by using the previously constructed regression model. The area under curve (AUC) was applied to evaluate the ability of a specific metabolite to differentiate test treatments, with an AUC value closer to 1 indicating that this metabolite was more reliable in diagnosing and differentiating treatments (AS gradients). Methionine (Met) was screened as the best signature metabolite for differentiating the AS gradient and its AUC value was 0.97, while 5-methylcytosine (5mC), with an AUC value of 0.94, could also be applied as a signature metabolite (Additional file 1: Fig. S2c), indicating that Met and 5mC responded most dramatically to AS addition. Met was readily activated by ATP to produce S-adenosylmethionine (AdoMet), which was the main methyl donor. 5mC and 3mA implied that a change in DNA methylation level may have occurred. The MS signal intensity could characterize intracellular metabolite content. The relative signal intensity of Met and AdoMet were significantly up-regulated in T3 relative to T1 (Fig. 2f), which corresponded to the ROC analysis results. 1-Methylthymine (1mT), 5-methylcytosine (5mC), 3-methyladenine (3mA), and 7-methylguanine (7mG) could serve as DNA methylation markers, where 5mC and 3mA were significantly up-regulated in T3 relative to T1, and 7mG was relatively up-regulated as well, while 1mT was not detected (Fig. 2g, Additional file 2: Dataset 1). It appeared that the increase in Met and AdoMet might be the trigger for the increase in 5mC and 3mA. The above results implied that AS-induced up-regulation of the lignocellulolytic response might be mainly through assimilation to Met, and this process might include changes in DNA methylation.

To investigate whether the increase in intracellular Met favored straw utilization, we increased the intracellular Met content by overexpressing the key enzyme in Met synthesis, 5-methyltetrahydrofolate-homocysteine methyltransferase gene (*ThmetH*, KEGG ID: K00549, GENE ID: A1A104551.1, NCBI ID: KKP03877.1), and obtained the strain OE-*ThmetH*. OE-*ThmetH* was cultured on MM + straw for 4 days (Fig. 2h), and its intracellular Met content (26.3 μmol·g^−1^) was significantly higher than that of WT (21.2 μmol·g^−1^), while the biomass and FPA of OE-*ThmetH* (2.26 × 10^5^ copy·g^−1^, 1.89 U·g^−1^) were also significantly up-regulated relative to WT (1.88 × 10^5^ copy·g^−1^, 1.44 U·g^−1^) (Fig. 2i, Additional file 1: Fig. S2d). The trend of EG and Xylanase activities was the same as that of FPA (Additional file 1: Fig. S6c) The above results indicated that up-regulation of intracellular Met facilitated the lignocellulolytic response of *T. harzianum*. Thus, the promotional effect of AS might be achieved mainly through assimilation to Met, and the process of Met promoting lignocellulosic response may involve inducing DNA methylation level changes.

### WGBS revealed that ammonium-sulfate addition induced methylation of *ThgsfR*2 promoter

**Fig. 3:**
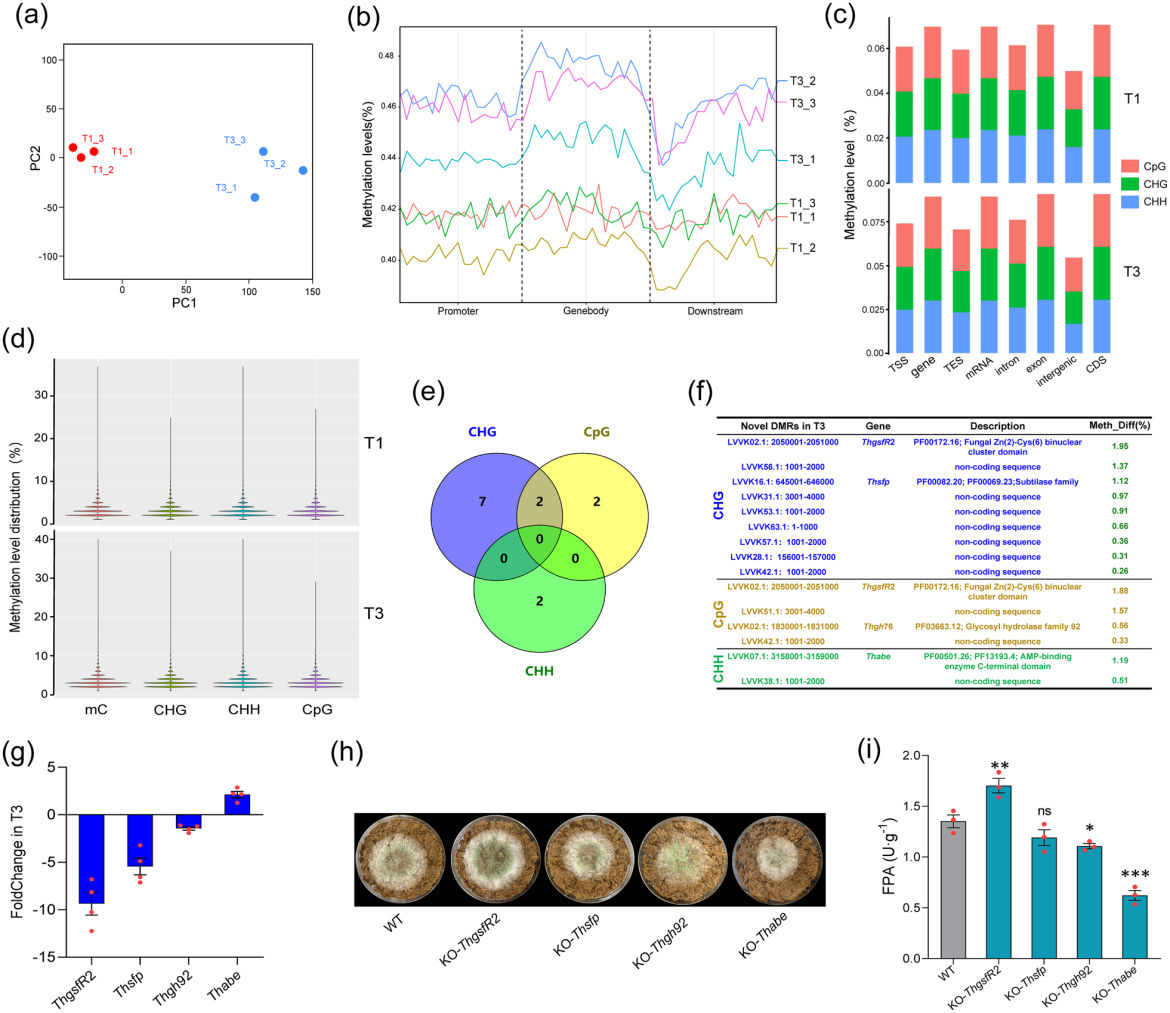
WGBS revealed that ammonium-sulfate assimilates induced the upregulation of DNA methylation level. **a** PCA demonstrated intra-group reproducibility and inter-group variability for T1 and T3. **b** Profiling analysis divided the region into 20 bins, and the methylation level in each bin reflected the trend of methylation level in the genome region. **c** Mean methylation levels of CG, CHG, and CHH type sites in exon, intron, UTR, and intergenic gene regions for each sample, with the horizontal coordinate being the genomic element type and the vertical coordinate indicating the methylation level, and the colors (red, green, and blue) indicated the mean methylation levels of the three types of sites. **d** Violin plots showed the numerical distribution of CpG, CHG, and CHH methylation levels for T1 and T3, with the vertical coordinate indicating the methylation level, and the width of each violin represented the number of points that were at that methylation level. **e** The Venn showed the distribution of novel DMRs of T3 and the number in CpG, CHG, and CHH types. **f** The location of the novel DMRs of T3 in chromosome fragment and its gene function. **g** FoldChange of the genes that were associated with the novel DMRs in T3 relative to T1. **h** Growth comparison for the mutants and WT on MM + straw. **i** FPA of the mutants and WT grown on MM + straw at 28 °C for 4 days. Bars represent mean ± SEM, with n = 3/4 biological repeats; red dots resemble values from individual experiments. Student’s *t*-testing was conducted in (**i**). *significant difference to WT at two-tailed *P* = 0.017 (**i**, KO-*Thgh*92); **significant difference to WT at two-tailed *P* = 0.002 (**i**, KO-*ThgsfR*2); ***significant difference to WT at two-tailed *P* = 0.000 (**i**, KO-*Thabe*); ns = no statistical difference to WT at two-tailed

**Fig. 4:**
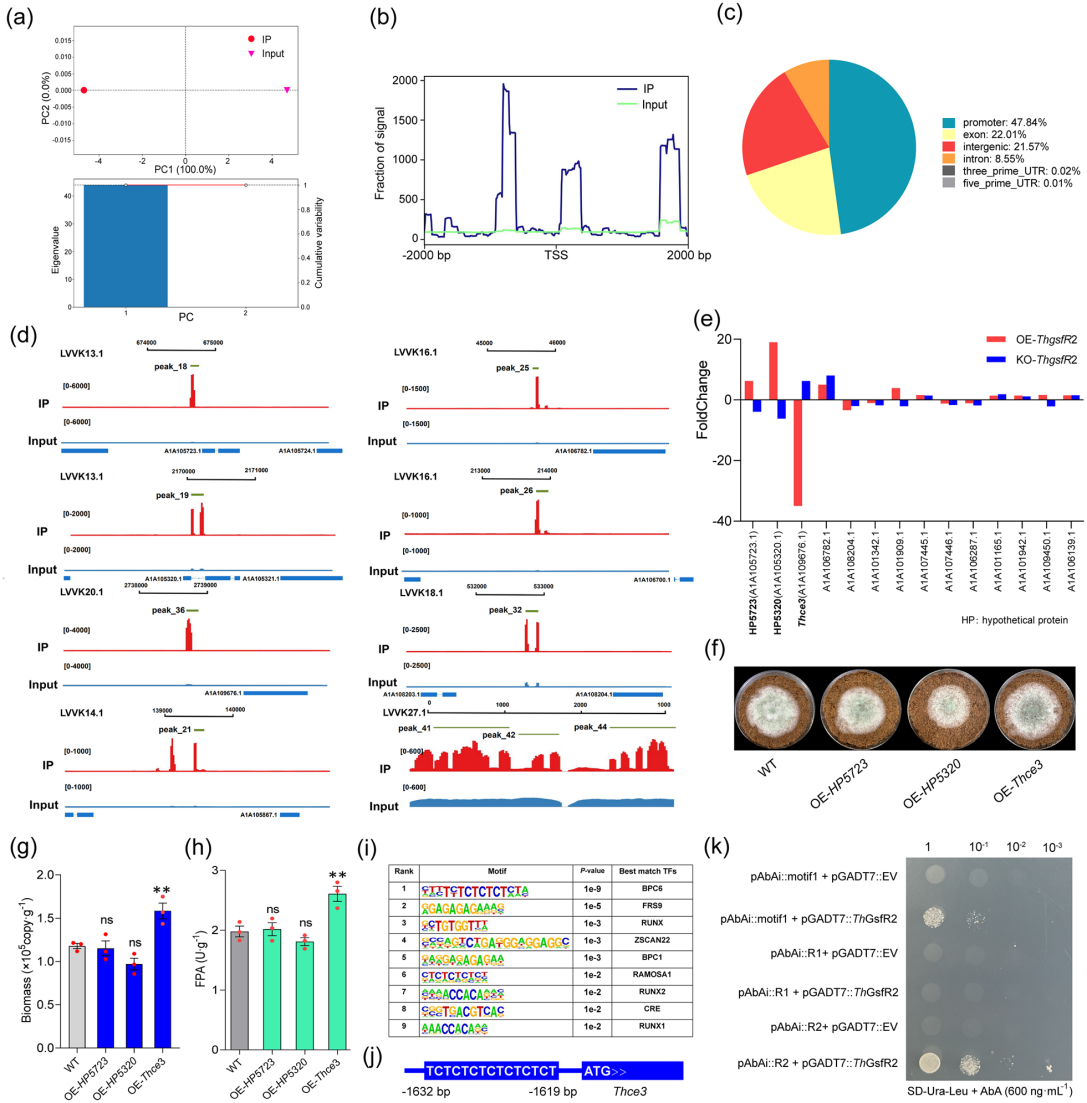
ChIP-seq identified the downstream genes regulated by the zinc finger transcription factor *Th*GsfR2. **a** PCA showed the difference in reads distribution between IP and Input. Notably, after data dimensionality reduction, PC1 has 100% interpretation on eigenvalue and cumulative variability. **b** The peak plot showed the distribution of peak reads of IP and Input. **c** Venn showed the distribution of peak reads over the genome functional elements. **d** The IGV visualization showed the location in genome of the 10 peaks with the highest enrichment of *Th*GsfR2, and the red column was IP, the blue column was Input, and column height indicated the signal intensity, data range was shown inside the “[]” on the left, the proximity genes for peaks were shown on the bottom. **e** Expression level FoldChange of the genes corresponding to peaks in OE-*ThgsfR*2 and KO-*ThgsfR*2 were compared to WT, note that we excluded the peaks located in non-gene functional regions. **f** Growth of OE-*HP5723*, OE-*HP5320*, OE-*Thce*3 and WT on MM + straw. **g** Biomass of OE-*HP5723*, OE-*HP5320*, OE-*Thce*3 and WT grown at 28 °C for 4 days. **h** FPA of OE-*HP5723*, OE-*HP5320*, OE-*Thce*3 and WT. **i** Motif analysis of all peaks using *Homer* yielded 10 *Th*GsfR2 binding motifs, ranked according to scoring, the last column of table showed the transcription factors predicted for motifs using *JASPAR*. **j** The conserved sequence “TCTCTCTCTC” of motif1 was presented in *Thce*3 promoter with 50-fold enrichment. **k** DNA–Protein interactions between *Th*GsfR2 and motif1, *Thce*3 promoter region R1 (−1000 to −1 bp), and R2 (−2000 to −1001 bp) were verified by Y1H assay. Bait-reporters (pAbAi::motif1, pAbAi::R1, and pAbAi::R2) could not grow in SD medium without uracil (SD-Ura) containing Aureobasidin A (AbA, 600 ng mL^−1^); pAbAi::motif1 + pGADT7::*Th*GsfR2 co-transformant and pAbAi::R2 + pGADT7::*Th*GsfR2 co-transformant could grow on SD-Ura-Leu containing AbA (600 ng mL^−1^), pAbAi::R1 + pGADT7::*Th*GsfR2 co-transformant could not grow on that medium. Bars represent mean ± SEM, with n = 3 biological repeats; red dots resemble values from individual experiments. Student’s *t*-testing was conducted in (**g**, **h**), **significant difference to WT at two-tailed *P* = 0.002 (**g**, OE-*Thce*3), 0.004 (**h**, OE-*Thce*3); ns = no statistical difference to WT at two-tailed *P* = 0.789 (**g**, OE-*ThHP5723*), 0.265 (**g**, OE-*ThHP5320*), 0.807 (**h**, *ThHP5723*), 0.077 (**h**, OE-*ThHP5320*)

Based on the metabolomics analysis results, multiple methylated metabolites were significantly up-regulated with AS addition. Noteworthy, 5mC was also screened as signature metabolite, implying that AS addition might induce a change in DNA methylation levels. Whole genome bisulfite sequencing (WGBS) was performed to evaluate the DNA methylation changes after AS addition. PCA demonstrated a good intra-group reproducibility and inter-group variability in T1 and T3 (Fig. 3a). By combining with NGS, the conversion rate of cytosine (C) to uracil (U) for each sample was counted, and the result indicated that T3 had higher DNA methylation levels in the promoter, genebody, and terminator than that in T1 (Fig. 3b), which validated the previous results obtained in metabolomics that 5mC, 3mA, and 7mG were up-regulated in T3. The average methylation levels of gene elements including exon, intron, TSS, intergenic, etc. on the DMRs of CpG, CHG, and CHH types were determined, which could show the effect of DMRs on gene expression (Fig. 3c). The numerical distribution of CpG, CHG, and CHH methylation levels of samples were counted and plotted as violins, in which the vertical coordinates indicated the methylation levels and the width of each violin represented how many points were at that methylation level (Fig. 3d). By analyzing the changes in DMRs methylation levels of GpG, CHG, and CHH types, we found that 4 GpG type DMRs were not present in T1 but were identified in T3, while 2 GHH type DMRs and 9 CHG type DMRs were similarly not present in T1 but were identified in T3. The Venn showed that the DMR in the chromosome fragment LVVK02.1 (2,050,001–2,051,000 bp) and LVVK42.1 (1001–2000 bp) was existed in GpG and CHG (Fig. 4e, Additional file 3: Dataset 2). By comparing the sequences of these 13 newly appeared DMRs in T3, we found that most DMRs were located in the beginning regions of chromosome fragments, such as LVVK31.1 (3001–4000 bp) and LVVK56.1 (1001–2000 bp), and LVVK63.1 (1–1000 bp), etc., which mostly were non-coding sequences. We screened DMRs located in gene transcriptional functional regions and compared them to their corresponding gene, the DMR located in LVVK02.1 (2,050,001–2,051,000 bp), which was the functional region of a zinc finger transcription factor gene (*ThgsfR*2, GENE ID: A1A109863.1, NCBI ID: OPB38861.1), which encoded a protein with high homology to the griseofulvin synthesis regulator GsfR2. The subtilase family protein gene (*Thsfp*, GENE ID: A1A100688.1, NCBI ID: OPB45967.1), glycosyl hydrolase family 92 protein gene (*Thgh*92, GENE ID: A1A100345.1, NCBI ID: OPB47137.1), and AMP-binding enzyme gene (*Thabe*, GENE ID: A1A100950.1, NCBI ID: OPB45998.1) were also included (Fig. 3f). DNA methylation in open reading frame (ORF) or promoter commonly affected transcription, we performed qPCR on the 4 genes, and result showed that the transcription levels of *ThgsfR*2 (9.4-folds) and *Thsfp* (5.4-folds) were down-regulated in T3 relative to T1, no significant Foldchange for *Thgh*92 (1.5-folds) and *Th*abe (2.1-folds) (Fig. 3g). To reveal the effect of down-regulation of these genes on lignocellulosic response of *T. harzianum*, these 4 genes were knocked out and obtained the mutants KO-*ThgsfR*2, KO-*Thsfp*, KO-*Thgh*92, and KO-*Thabe*. The growth of KO-*ThgsfR*2 on MM + straw was better than WT, while KO-*Thgh*92 and KO-*Thabe* were worse than WT, and the difference between KO-*Thsfp* and WT were not significant (Fig. 3h). The biomass and FPA were increased in KO-*ThgsfR*2 (2.52 × 10^5^ copy g^−1^, 1.71 U g^−1^) and decreased in KO-*Thgh*92 (1.22 × 10^5^ copy g^−1^, 1.11 U g^−1^) and KO-*Thabe* (0.45 × 10^5^ copy g^−1^, 0.62 U g^−1^) relative to WT (× 10^5^ copy g^−1^, 1.35 U g^−1^), while these parameters of KO-*Thsfp* (1.59 × 10^5^ copy g^−1^, 1.19 U g^−1^) was not significantly different from WT (1.81 × 10^5^ copy g^−1^, 1.35 U g^−1^) (Fig. 3i, Additional file 1: Fig. S3b). The trend of EG and Xylanase activities was the same as that of FPA (Additional file 1: Fig. S6d). Since the growth of KO-*Thabe* on PDA was also affected, the drastic decrease in straw utilization capacity could be due to the absence of *Thabe* affecting basal metabolism (Additional file 1: Fig. S3a). These results suggested that AS promoted lignocellulose utilization of *T. harzianum* involved indirectly inducing the methylation of *ThgsfR*2 promoter. Therefore, *Th*GsfR2 might be a negative regulator of lignocellulosic responses in addition to regulating griseofulvin expression, and the methylation contributed to the release of lignocellulolytic response while inhibiting secondary metabolism. Similarly, the regulator LaeA played a critical role in regulating the biosynthesis of aflatoxin, penicillin, and lovastatin, controlling cellulase synthesis [43, 44]. Ypr1 regulates the yellow pigment sorbicillin biosynthesis in *T. reesei*, and the knockout strain of YRP (KO-*Trypr*1) showed a decrease in secondary metabolites and an increase in biomass, notably, the cellulase gene (*Trcbh*1, *Trbgl*, *Trbxl*1) and xylanase gene *Trxyn*1 were up-regulated [45]. Our results also indicated that the inhibition of griseofulvin synthesis was beneficial for the up-regulation of biomass and total enzyme activity in *T. harzianum*, suggesting that inhibition of secondary metabolism favored the up-regulation of CAZymes.

Chemical or biological factors could induce an increase in DNA methylation levels [46, 47], but how the components of DNA methylation are recruited to genome-specific sites remains to be investigated. Factually, AdoMet was one of the intracellular inducers for DNA methylation [48, 49], and significantly upregulated when AS was added, which might be the inducer for methylation of *ThgsfR*2 promoter. The exogenous AdoMet addition was reported to promote cellulase synthesis in *Penicillium oxalicum* [50]. Notably, most of the novel DMRs induced by AS occurred in intergenic regions. The intergenic regions could also regulate cellular behaviors by encoding microRNAs [51–53]. The Dicer-dependent microRNAs (miRNAs) and various small interfering RNAs (siRNAs), such as exo-siRNAs, endo-siRNAs, and natsiRNAs [54] can regulate the expression of plant cell wall degrading enzyme (PCWDE) genes or secondary metabolism [55]. Thus, whether the AS-induced methylation in intergenic regions was equally involved in the regulation of the lignocellulolytic response by affecting microRNA expression deserved further investigation.

### ChIP-seq uncovered the downstream genes regulated by *Th*GsfR2

To reveal which gene was negatively regulated by *Th*GsfR2, we performed Chromatin immunoprecipitation (ChIP). ChIP was an in-situ and in-vivo assay that can reveal the downstream genes regulated by *Th*GsfR2. The strain *Th*GsfR2-His was constructed by adding His-tag to C-terminus of *Th*GsfR2 and validated by western blotting (Additional file 1: Fig. S4a). *Th*GsfR2-His was incubated at 28 °C for 4 days, after formaldehyde cross-linking and ultrasonic fragmentation of chromatin (Additional file 1: Fig. S4b), *Th*GsfR2 and DNA complexes were specifically recognized and precipitated by ChIP-class His-tag antibody and protein A/G beads, and then decross-linked and recovered to yield pure DNA fragments. The products of IP and Mock (IgG) were sequenced and mapped with the *T. harzianum* genome. PCA showed a significant difference between IP and Mock (IgG), suggesting that IP treatment has effective nucleic acid precipitation (Fig. 4a). The transcription start site (TSS) proximal (0–2 kb) was associated with a specific transcriptional regulatory function, and the reads distributed within the TSS proximal were counted; the peak plot showed the distribution and enrichment of all peaks near the TSS proximal (Fig. 4b). The number of peaks on the annotated genomic structural elements (intergenic, promoter, 5’UTR, exon, intron, 3’UTR) was counted, and their enrichment and distribution characteristics on each element were also counted. Peaks were most distributed on promoter, accounting for 47.84%, followed by exon (22.01%), and 30.12% in non-functional areas (intergenic and intron) (Fig. 4c). IGV visualization showed the distribution on genome of the 10 peaks with the highest enrichment folds (Fig. 4d).

After sequencing and genomic alignment of the IP and Input products, 44 peaks with differential enrichment folds (folds > 2) were obtained (Additional file 4: Dataset 3). Combining the peaks on gene elements, the peaks on intergenic and intron were filtered out, and 14 genes were confirmed to be potentially regulated by *Th*GsfR2. The mutant OE-*ThgsfR*2 (56.8-fold up-regulated) and KO-*ThgsfR*2 were constructed by overexpressing and knocking out *ThgsfR*2 to confirm the downstream gene regulated by *Th*GsfR2. The transcription level changes of the 14 genes in OE-*ThgsfR*2 and KO-*ThgsfR*2 relative to WT were quantified by qPCR. The peaks mapped genes with a low fold enrichment (folds < 10) did not show a significant Foldchange with up-regulation (OE) and down-regulation (KO) of *ThgsfR*2. The transcription levels of nucleotide binding hypothetical protein THAR02_03409 gene (*HP5723*, GENE ID: A1A105723.1, NCBI ID: OPB42089.1) and hypothetical protein TRIVIDRAFT_215543 gene (*HP5320*, GENE ID: A1A105320.1, NCBI ID: OPB42540.1) displayed a significant linear correlation with the *ThgsfR*2 expression levels. The carbohydrate esterase family 3 protein gene (*Thce*3, GENE ID: A1A109676.1, NCBI ID: OPB38703.1) was 34.9-fold down-regulated in OE-*ThgsfR*2 and 14.2-fold up-regulated in KO-*ThgsfR*2 (Fig. 4e), which suggested a negative regulation of *Th*GsfR2 on *Thce*3.

### Up-regulation of *Thce*3 facilitated the lignocellulosic response of *T. harzianum*

Since methylation or knockout of *ThgsfR*2 favored lignocellulolytic response, this suggested that *Th*GsfR2 repressed some genes involved in lignocellulolytic response, and *HP5723*, *HP5320*, and *Thce*3 might be the repressed genes. The effect of these genes on the lignocellulose utilization capacity of *T. harzianum* was investigated by overexpression and obtained the mutants OE-*HP5723*, OE-*HP5320*, and OE-*Thce*3. These mutants were cultured at 28 ℃ for 4 days (Fig. 4f) and the biomass and FPA were also evaluated. The biomass and FPA of OE-*Thce*3 (1.58 × 10^5^ copy g^−1^, 2.61 U g^−1^) was significantly greater than that of WT (1.18 × 10^5^ copy g^−1^, 1.98 U·g^−1^), and these parameters of OE-*HP5723* (1.15 × 10^5^ copy g^−1^, 2.01 U g^−1^) and OE-*HP5320* (0.97 × 10^5^ copy·g^−1^, 1.81 U g^−1^) were not significantly different from WT (Fig. 4g, h). The same trends were obtained in EG and Xylanase activities (Additional file 1: Fig. S6e). These results suggested that of all the genes that changed significantly with *Th*GsfR2 expression level, only *Thce*3 was directly implicated in lignocellulose utilization of *T. harzianum*, which was reasonable because *Thce*3 could act directly on polysaccharides and belonged to the CAZymes family. Overexpression of the two hypothetical proteins (*HP5723* and *HP5320*) did not promote the growth of *T. harzianum* on straw, suggesting that they were not lignocellulose utilization related genes, and since their transcription levels linearly correlated with the expression level of *Th*GsfR2, HP5723, and HP5320 might have belonged to the griseofulvin synthesis pathway.

The binding sequence preference of *Th*GsfR2 could be obtained by motif analysis on all peaks. The table ranked the motifs sequence characteristics of the transcription factor *Th*GsfR2 based on Homer analysis and scoring, and these motifs were predicted for the reported transcription factors through the Jasper transcription factor database (Fig. 4i). Notably, the conserved sequence “TCTCTCTCTC” of motif1 with the highest confidence was found in the *Th*ce3 promoter region (−1632 to −1619 bp), correspondingly, peak_36 was coincidentally located at about −1632 to −1619 bp upstream of *Thce*3 (A1A109676.1) (Fig. 4j). The protein-DNA interactions between *Th*GsfR2 and motif1 was verified by yeast one-hybrid assay (Y1H) and the interactions between *Th*GsfR2 and the *Thce*3 promoter region1 (R1: −1000 to −1 bp) and region 2 (R2: −2000 to −1001 bp) (Additional file 1: Fig. S4c) were also verified. Notably, R2 contained motif1. The result indicated that *Th*GsfR2 exhibited protein-DNA interaction with motif1, strong interaction with R2, and almost no interaction with R1 (Fig. 4k). Combined with the qPCR result, it could be extrapolated that *Th*GsfR2 negatively regulated *Thce*3 through competitive binding with RNA polymerase. Therefore, methylation of *Th*GsfR2 gene facilitated the reduction of its repression on *Thce*3 thereby releasing the expression potential of *Th*CE3.

### AS induced up-regulation of multiple CEs significantly enhanced substrate deacetylation of *T. harzianum*

**Fig. 5:**
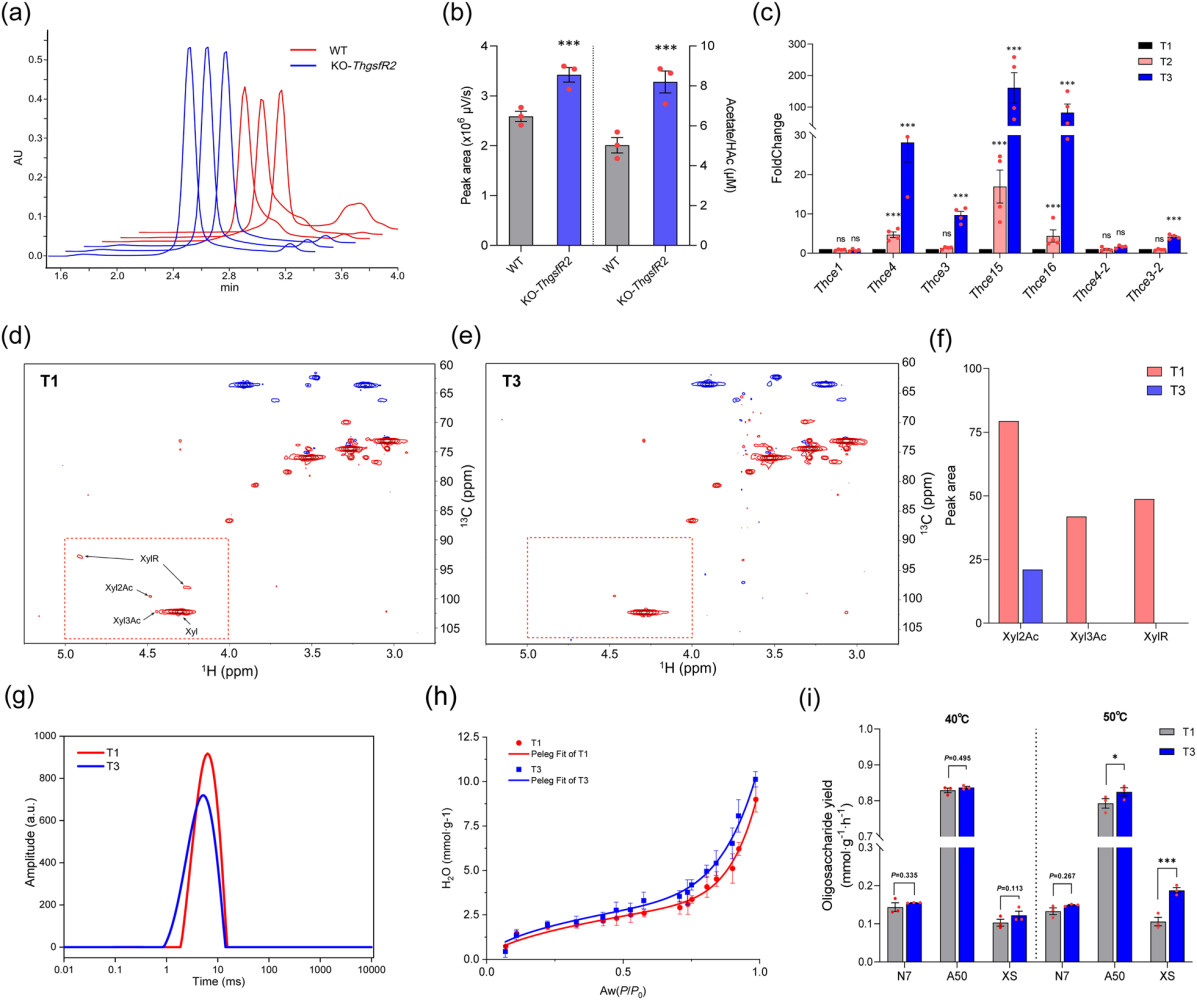
Ammonium-sulfate induced the up-regulation of multiple CEs, which in turn improved glycosidic bond accessibility through xylan deacetylation. **a** HPLC results of acetate after 10-day liquid fermentation of WT and KO-*ThgsfR*2. The red line was WT treatments, and blue line was KO-*ThgsfR*2 treatments, note that the acetate mainly comes from acetyl-groups. **b** Peak area and concentration of acetate for WT and KO-*ThgsfR*2 treatments. Concentration was obtained by peak areas and standard regression equation. **c** Expression levels quantification of CEs genes with AS gradient (T1, T2, T3). qPCR was performed on the 7 identified CEs genes and multiple CE genes were up-regulated with the AS gradient. **d** HSQC spectrum of extracted xylan from WT-treated straw under T1 condition for 15 days. The signals from Xyl2Ac and Xyl3Ac could be detected. **e** HSQC spectrum of extracted xylan from WT-treated straw under T3 condition for 15 days, The signal peak area of Xyl2Ac was reduced and Xyl3Ac has no detectable signal. **f** Quantitative results of Xyl2Ac, Xyl3Ac, and XylR peak areas in the T1 and T3 NMR spectrum, which can indicate the change in acetyl content with the AS addition. The peak areas of Xyl2Ac, Xyl3Ac, and XylR were all decreased in T3 relative to T1. **g** T2 relaxation spectrum of T1 and T3. Low-field NMR determined the spin–spin relaxation time (*T*_2_) of straw after hyphal treated on T1 and T3 conditions for 15 days. **h** WSI of hyphae-treated (15 days) straw under T1 and T3 conditions, noting that the Peleg modeled WSI belonged to type II isotherm. **i** Degradability comparison of hyphae-treated (15 days) straw under T1 and T3 conditions. GHs (N7, A50, and XS) were used to hydrolyze hyphae-treated straw, and the affinity efficiency of GHs for polysaccharide glycosidic bonds was compared by determining the reducing sugars (40 °C and 50 °C reaction for 20 min). Note that this could compare the effects of different acetylation levels on GHs binding glycosidic bonds. Bars represent mean ± SEM, with n = 3 or 4 biological repeats; red dots resemble values from individual experiments. Student’s *t*-testing was conducted in (**b**, **c**, **i**), *significant difference to T1 at two-tailed *P* = 0.002 (**i**, T3: A50-50 ℃). ***significant difference to WT at two-tailed *P* = 0.000 (**b**, KO-*ThgsfR*2: peak area), 0.000 (**b**, KO-*ThgsfR*2: acetate). ***significant difference to T1 at two-tailed *P* = 0.000 (**i**, T3: XS-50 ℃). ns = no statistical difference to T1 at two-tailed *P* = 0.335 (**i**, T3: N7-40 ℃), 0.495 (**i**, T3: A50-40 ℃), 0.113 (**i**, T3: XS-40 ℃), 0.267 (**i**, T3: N7-50 ℃)

Previous experiments have shown that AS addition eventually released the expression of *Thce*3 by inducing methylation of *ThgsfR*2 promoter, which in turn increased the lignocellulose utilization efficiency of *T. harzianum*. The main function of CEs was to deacetylate polysaccharides [56], and the removed acetyl groups were detectable [40]. The KO-*ThgsfR*2 and WT were subjected to liquid fermentation for 7 days, and 1 mL centrifuged supernatant was used to detect the acetate in the fermentation broth by high-performance liquid chromatography (HPLC). The results showed that the signal intensity of acetate peak of KO-*ThgsfR*2 treatment was significantly greater than that of WT and had good reproducibility (Fig. 5a). By counting the peak area, acetate content in treatments was obtained by standard regression equation (Additional file 1: Fig. S5a, b). The acetate content of KO-*ThgsfR*2 treatment was significantly greater than that of WT treatment (Fig. 5b), which further suggested that there was a negative regulatory of *Th*GsfR2 on *Thce*3. AS induced up-regulation of *Thce*3 enlightened us to quantify the expression levels of other CE family genes that were annotated in *T. harzianum*. After extracting hyphae RNA grew under AS gradients (T1, T2, and T3) and reversing transcription, the expression of the CE family genes was quantified by qPCR. Excitingly, except for *Thce*1 (GENE ID: A1A101843.1, NCBI ID: OPB45480.1), including *Thce*3, *Thce*3-2 (GENE ID: A1A102051.1, NCBI ID: OPB45653.1), *Thce*4 (GENE ID: A1A101972.1, NCBI ID: OPB45589.1), *Thce*4-2 (GENE ID: A1A108380.1, NCBI ID: OPB39263.1), *Thce*15 (GENE ID: A1A103020.1, NCBI ID: OPB43780.1), and *Thce*16 (GENE ID: A1A106549.1, NCBI ID: OPB27301.1) all showed a significant up-regulation linearly with AS gradient, even the foldchanges of *Thce*15 and *Thce*16 greater than *Thce*3 (Fig. 5c). CE3 and CE4 defined as acetyl xylan esterase mainly responded to catalyze *O*-2-deacetylation and O-3-deacetylation of xylose residues, while CE15 mainly deacetylating the glucuronic acid. CE16 was a non-specific acetylesterase, which could deacetylate oligomeric xylan [57].

It follows that AS addition induced the up-regulation of multiple CEs, which facilitated the deacetylation of substrate. Gramineae such as rice have a high degree of polysaccharide acetylation, leading to GHs could not effectively binding the glycosidic bonds, which greatly limited the efficiency of straw saccharification [15, 58]. CEs could assist GHs in efficiently hydrolyzing polysaccharides through deacetylation. Usually, acetylation in rice polysaccharides occurred mainly on xylan [59], hence the change in xylan acetylation level of *T. harzianum* treated straw was examined by 2D nuclear magnetic resonance (2D NMR). *T. harzianum* was inoculated in the straw medium under T1 and T3 (AS addition) conditions at 28 °C for 15 days, and xylan was extracted from the straw after removing hyphae. The extracted xylan was dissolved using DMSO-d_6_ for heteronuclear single quantum coherence (HSQC) assay. The HSQC spectra showed that the signal peak of 2-*O*-Acetyl-Xylosyl residues (Xyl2Ac: 99.71 ppm, 4.46 ppm) was reduced in the T3 spectrum compared with T1, and 3-*O*-Acetyl-Xylosyl (Xyl3Ac: 102.11 ppm, 4.43 ppm) was even undetectable in the T3 spectrum. Other chemical modification residues (XylR: 92.89 ppm, 4.91 ppm; 98.11 ppm, 4.26 ppm) were also undetectable in the T3 spectrum (Fig. 5d, e). The peak areas of Xyl2Ac, Xyl3Ac, and XylR in T1 and T3 were counted, and the result indicated that AS was able to enhance the xylan deacetylation by inducing the up-regulation of CEs, and the deacetylation on 3-*O*-Acetyl-Xylosyl was more efficient (Fig. 5f).

### Lower acetylation level favored the hydrophilicity of polysaccharides

Generally, the relaxation time *T*_2_ could characterize the moisture affinity efficiency of polysaccharides, and a larger *T*_2_ indicated a higher water freedom [60]. The straw powder after hyphae treated was washed and centrifuged, and the samples of T1 and T3 were aliquoted and analyzed by low-field nuclear magnetic resonance (low-field NMR), and the *T*_2_ relaxation spectra showed that the *T*_2_ time of T3 was smaller than that of T1 (Fig. 5g; Additional file 5: Dataset 4), indicating that a smaller water freedom degree in T3, which further suggested that the straw decomposed by hyphae under T3 (AS addition) conditions has better hydrophilicity relative to T1.

Deacetylation not only removed the spatial barrier of glycosidic bonds but also exposed the hydroxyl group, thereby increasing the hydrophilicity of polysaccharides. The water sorption isotherms (WSI) could reflect the hydrophilicity of material. After drying, the water uptake of straw powder in different relative pressures (*P*/*P*_0_) was determined, and the isotherms were obtained by the Peleg modeling. The WSI of treatments (T1 and T3) exhibited the type II WSI. Notably, the WSI of T3 was above T1 (at the same relative pressure), indicating that the straw powder degraded by *T. harzianum* grown in T3 (AS addition) owned better hydrophilicity than T1 (Fig. 5h). This result indicated that the deacetylation of xylan has the advantage of improving the hydrophilicity of straw polysaccharides.

Glycoside hydrolases (GHs) need to be soluble in water to hydrolyze glycosidic bonds, so an increase in substrate hydrophilicity might improve the accessibility of GHs to glycosidic bonds, thereby increasing the hydrolysis efficiency. Therefore, we used commercial GHs (N7, A50, XS) to hydrolyze hyphae-treated straw powder, and the production of reducing sugars could characterize the digestibility of substrate. The result showed that at 40 °C, GHs hydrolyzed hyphae-treated straw powder under T3 conditions produced higher reducing sugars than T1, but the difference was not significant. The same result was obtained at 50 °C, and the difference between T1 and T3 reached a significant level (*P* < 0.05) in A50 and XS (Fig. 5h). These suggested that the increased hydrophilicity of polysaccharides favored the hydrolysis efficiency of GHs. The above experimental results indicated that AS addition facilitated the hydrophilicity increase of polysaccharides and the spatial barrier reduction of glycosidic bonds by inducing the up-regulation of CEs, which in turn improved the accessibility of glycosidic bonds to glycoside hydrolases.

## Conclusions

**Fig. 6:**
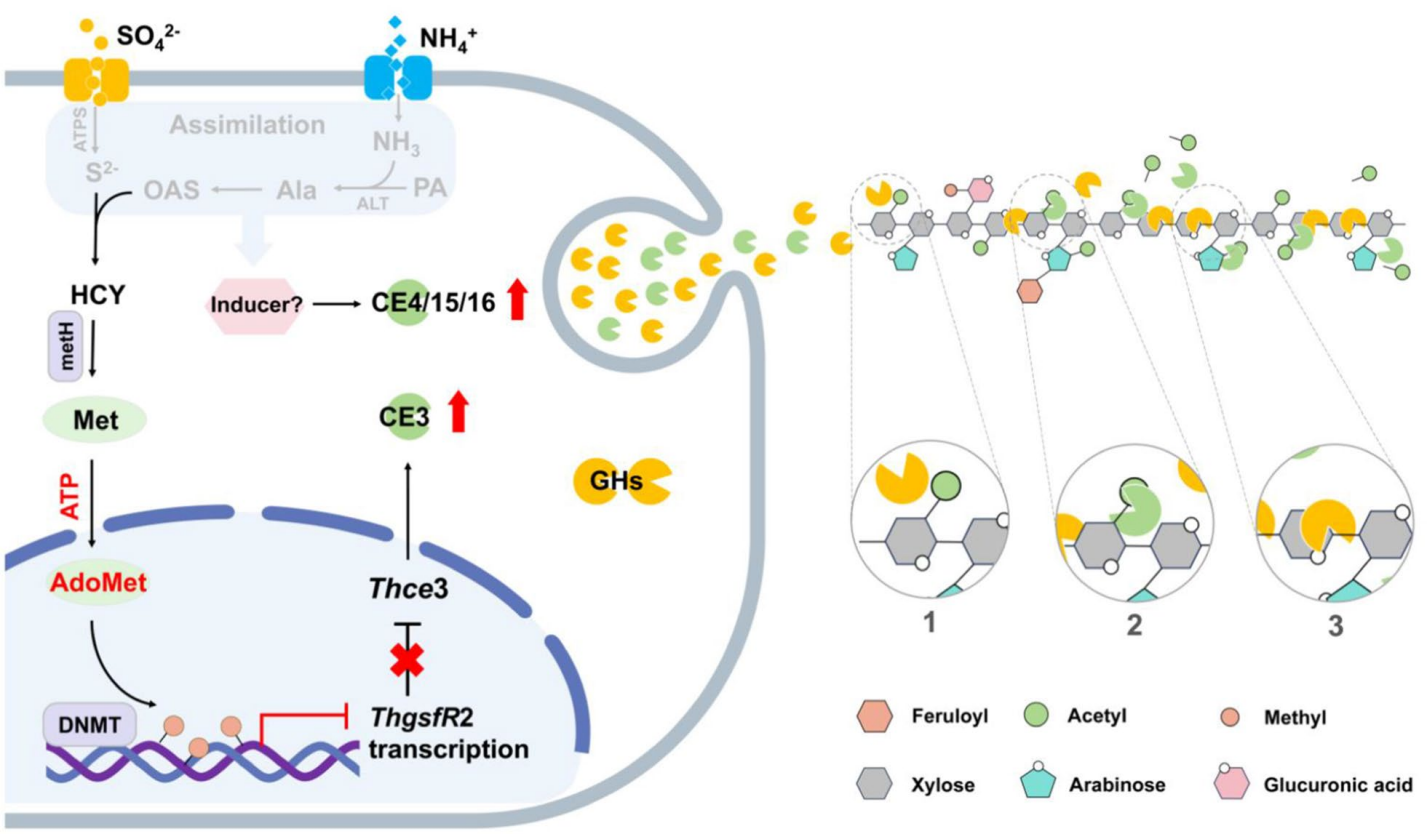
Schematic diagram of ammonium-sulfate induced up-regulation of multiple CEs and thereby increased glycosidic bond accessibility. Normally, the transcription factor *Th*GsfR2 inhibited *Th*CE3 expression by competitive binding of the functional region of *Thce*3 promoter. After the ammonium-sulfate addition, AS would be transported into the cell by SULTR and AMT, and ammonium ions were converted to NH_3_ by deprotonation, while SO_4_^2−^ was reduced into S^2−^ by ATPS. Pyruvate produced by glycolysis bound NH_3_ to produce alanine (Ala). Ala would be converted to O-acetyl homoserine (OAHS) by multi-enzyme catalysis. Subsequently, OAHS would be converted to homocysteine (HCY) catalyzed by the O-acetyl homoserine sulfhydrylase, and further catalyzed by 5-methyltetrahydrofolate-homocysteine methyltransferase (metH) to produce the terminal assimilates methionine (Met). Met was converted to AdoMet by ATP activation and induced methylation of the *ThgsfR*2 promoter, leading to transcriptional repression of *ThgsfR*2. This allowed *Thce*3 to be released from the repression of *Th*GsfR2, exhibiting a significant up-regulation of transcriptional level. In addition, multiple CEs were induced to be up-regulated by unknown inducers generated by AS assimilation. Up-regulation of CEs enhanced polysaccharide deacetylation, which in turn increased hydrophilicity and removed the spatial barrier of glycosidic bonds. **1**. Acetylation of xylose residues prevented glycoside hydrolases from glycosidic bonds. **2**. CEs catalyzed deacetylation of the xylose acetyl group. **3**. Deacetylation removed the spatial barrier of glycosidic bonds and increased the accessibility of the glycosidic bond to glycoside hydrolases

The lignocellulose decomposition promotional effect of ammonium-sulfate was mainly achieved through cellular assimilation to Met, which was activated by ATP into an active methyl donor (AdoMet), leading in turn to methylation of the *ThgsfR*2 promoter and an increase in DNA methylation level. Inhibition of *ThgsfR*2 by methylation released the expression of *Th*CE3, while multiple *Th*CEs were significantly up-regulated in response to the AS-inducing, thereby increasing the efficiency of substrate deacetylation. Deacetylation of polysaccharides improved hydrophilicity and removed the spatial barriers of glycosidic bonds hydrolysis, i.e., improved accessibility of glycosidic bonds (Fig. 6). Higher efficiency of glycoside hydrolases in hydrolyzing polysaccharides facilitated plant residues utilization and biochemicals conversion by *T. harzianum*. These results contributed to further understanding the complex CAZyme-producing regulatory network in fungi and the importance of carbohydrate esterases in plant residues biodegradation, which provide insights to improve fermentation efficiency and novel targets for metabolic engineering modification.

### Supplementary Information

**Additional file 1.** The supplemental results.

**Additional file 2.** The changes in relative content of intracellular metabolites identified by metabolomics.

**Additional file 3.** All differentially methylated regions (DNRs) in T1 and T3.

**Additional file 4. All Th** GsfR2 precipitated nucleic acid fragment information identified by ChIP-seq.

**Additional file 5.** The raw data for measuring *T2* relaxation time using low field NMR.

## Data Availability

All data generated during this study are included in this article and the additional files. Raw data are available on reasonable request.
