# Supplementary material for: Methionine inducing carbohydrate esterase secretion of Trichoderma harzianum enhances the accessibility of substrate glycosidic bonds
Source: Microb Cell Fact. 2024 Apr 26;23:120. doi: 10.1186/s12934-024-02394-1 (PMC11046756; doi:10.1186/s12934-024-02394-1)
Supplement: Supplementary file 1 — Additional file 1. The supplemental results. [file 12934_2024_2394_MOESM1_ESM.docx]

**Supplementary Materials For**

**Methionine inducing carbohydrate esterase secretion of *Trichoderma harzianum* enhances the accessibility of substrate glycosidic bonds**


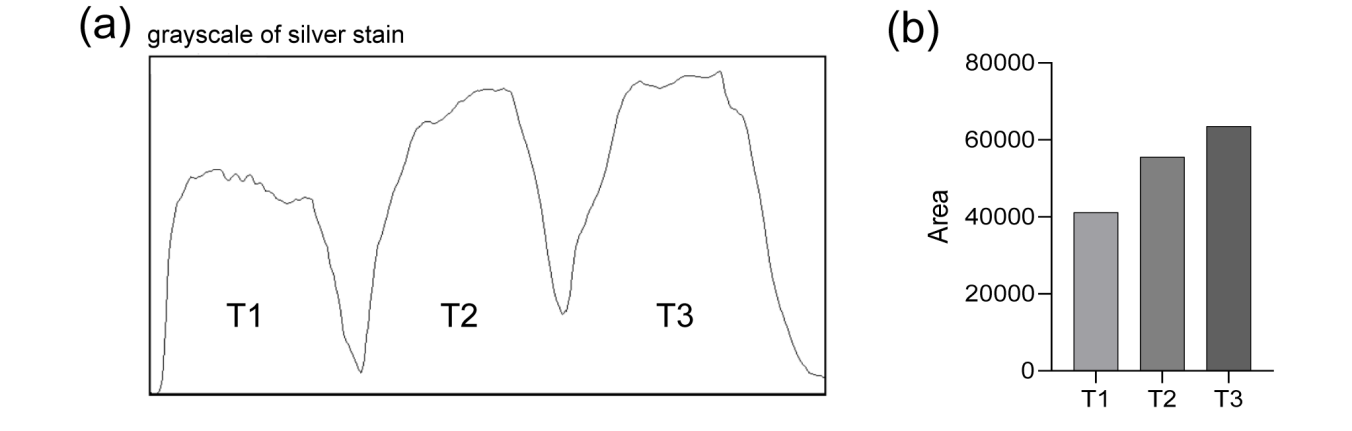


**Fig. S1 Grayscale quantification of silver-stained protein gels. (a)** The grayscale analysis of the silver staining result by *ImageJ* showed the protein lanes of T1, T2, and T3, the larger area represented the larger grayscale, which also indicated the higher extracellular protein content. **(b)** Datametric presentation of Fig. S1a. The smallest area of T1 indicated the lowest extracellular proteins content, and the largest area of T3 indicated the highest extracellular proteins content.


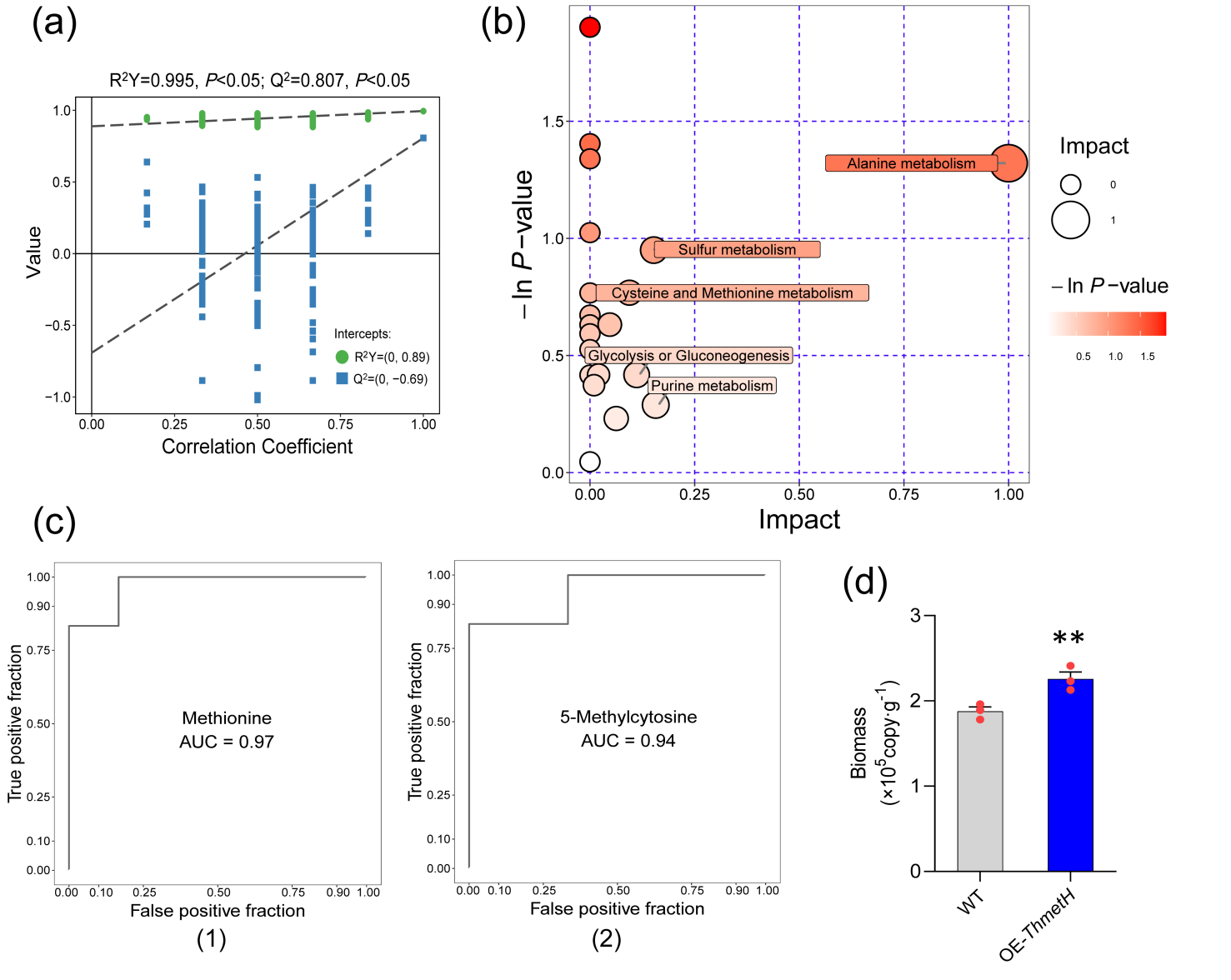


**Fig. S2 Model construction, ROC analysis, and pathway response analysis results of metabolomic. (a)** Correlation analysis of metabolites and categories. The validity of the OPLS-DA model indicated a high interpretability for categorical variables (R^2^Y = 0.995, *P* < 0.05) and high predictability (Q^2^ = 0.807, *P* < 0.05). **(b)** Bubble plot of pathway response. Alanine (Ala) metabolism responded most dramatically to AS addition and had the largest impact factor. Ala metabolism was the key process for ammonia assimilation, while Ala was also an important precursor for Met synthesis. This was followed by sulfur metabolism and methionine metabolism, which were closely related to the synthesis of Met, the primary assimilate of thionine. **(c)** ROC analysis of methionine and 5-methylcytosine. (c1) The AUC of methionine was 0.97 (close to 1), suggesting the drastic change in intracellular methionine content in T3 (AS added) relative to T1, which could be served as a signature metabolite. (c2) The AUC of 5-methylcytosine was 0.94 (close to 1), suggesting that 5-methylcytosine could also be served as a signature metabolite. **(d)** Biomass comparison of WT and OE-*ThmetH*. The strains were cultured on MM+ straw at 28°C for 4 days. Bars represent mean ± SEM, with n = 3 biological repeats; red dots resemble values from individual experiments. Student’s *t*-testing was conducted in (d). **signiﬁcant difference to WT at two-tailed *P* = 0.003 (d, OE-*ThmetH*).


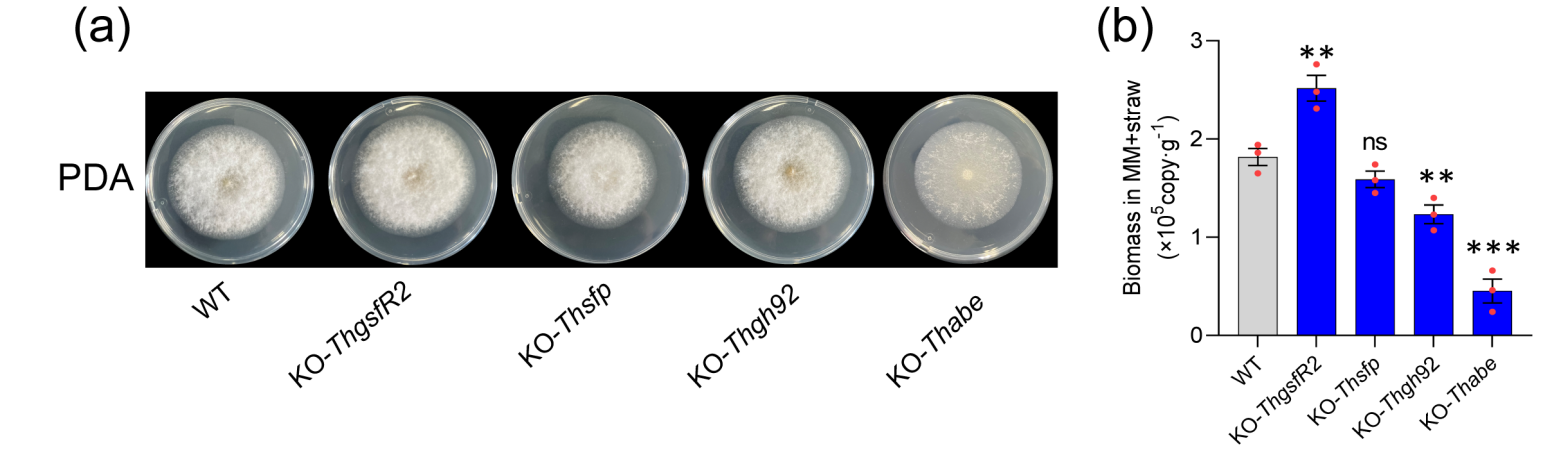


**Fig. S3 Effects of gene knockouts on basal metabolism. (a)** The growth of KO-*ThgsfR*2, KO-*Thsfp*, KO-*Thgh*92, and KO-*Thabe* on PDA. Notably, KO-*Thabe* grew significantly worse than WT on preferred carbon and nutrient-sufficient medium (PDA), suggesting that *Thabe* deficiency severely affected basal metabolism. (b) The biomass of KO-*ThgsfR*2, KO-*Thsfp*, KO-*Thgh*92, and KO-*Thabe* on MM+straw. *ThgsfR*2 deletion significantly facilitated lignocellulose. Bars represent mean ± SEM, with n = 3 biological repeats; red dots resemble values from individual experiments. Student’s *t*-testing was conducted in (b). **signiﬁcant difference to WT at two-tailed *P* = 0.002 (b, KO-*ThgsfR*2), 0.003 (b, KO-*Thgh*92). ***signiﬁcant difference to WT at two-tailed *P* = 0.000 (b, KO-*Thabe*). ns = no statistical difference to WT at two-tailed *P* = 0.159 (b, KO-*Thabe*).


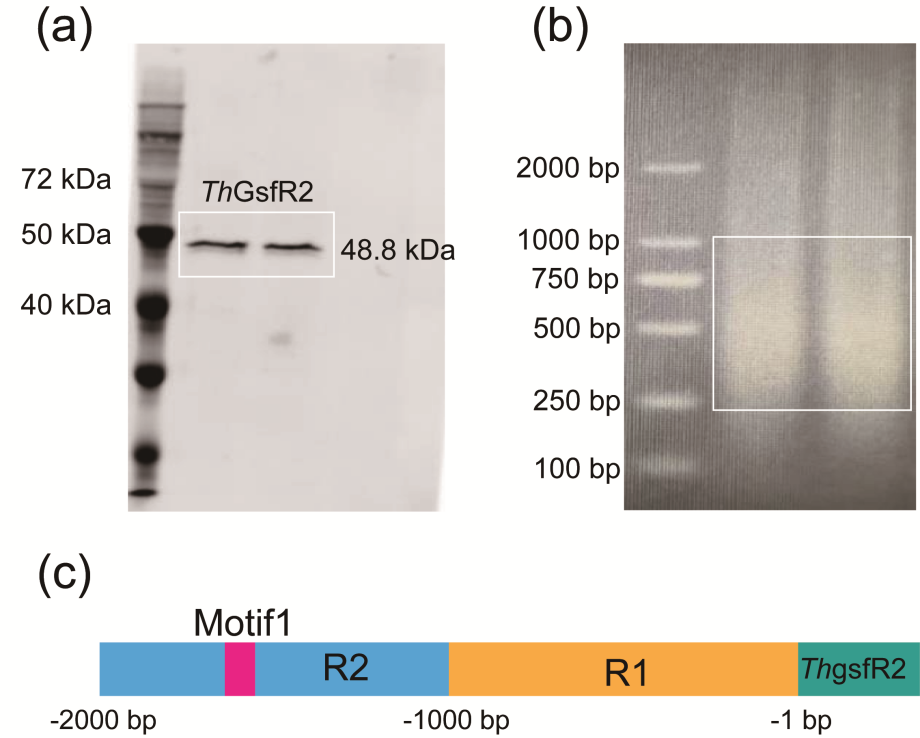


**Fig. S4 ChIP-related results and binding motif identification for *Th*GsfR2. (a)** Western blotting validation for *Th*GsfR2. *Th*GsfR2 was about 48.8 kDa; His-tag was added to the C-terminus. **(b)** DNA electrophoresis verified ultrasonic fragmentation of chromatin. The majority of formaldehyde cross-linked chromatin was fragmented to 200 bp - 1000 bp. (c) Distribution of fragments (R1, R2, motif1) in the ThgsfR2 promoter region, which were inserted into pAbAi in Y1H assay.


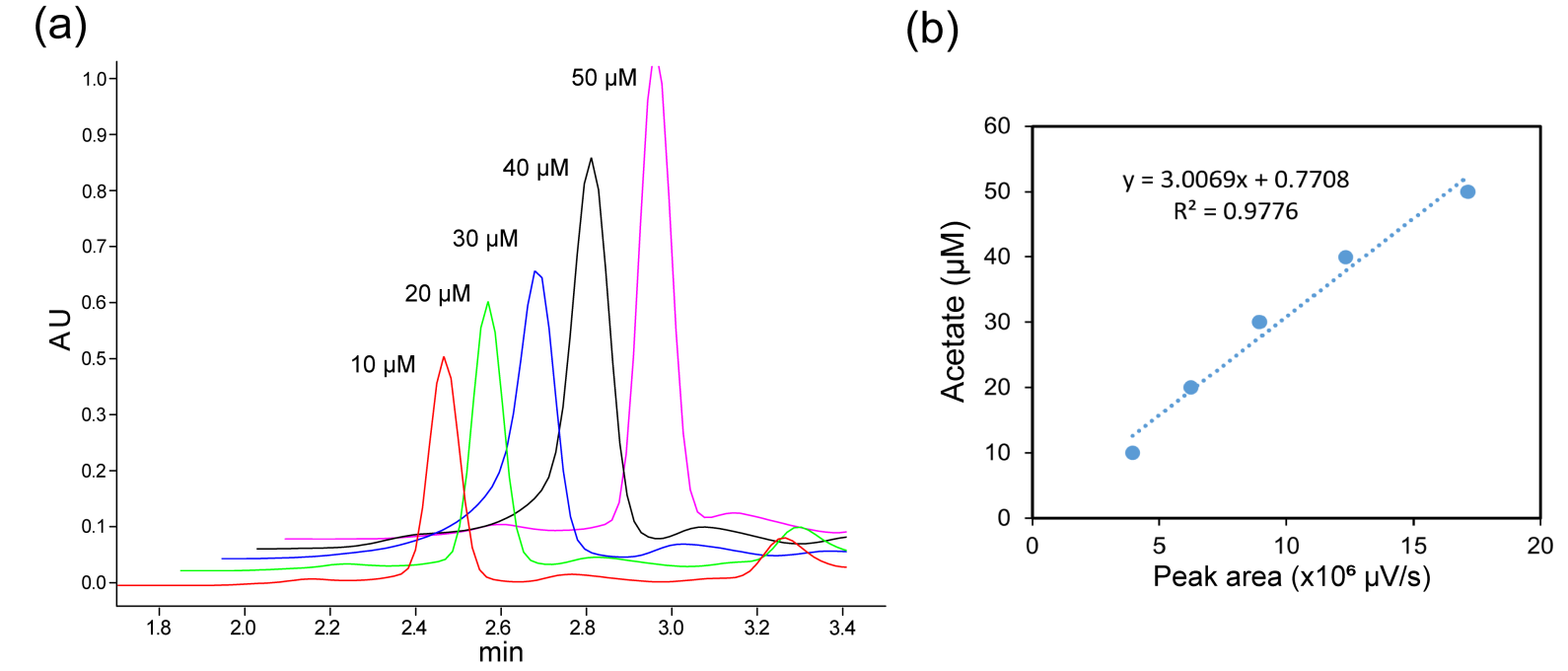


**Fig. S5 Standard regression equation for the acetate quantification in fermentation broths. (a)** Potassium acetate gradient (10 µM, 20 µM, 30 µM, 40 µM, 50 µM) solutions were used to make standard regression equations for acetate and liquid chromatographic peak areas. **(b)** The regression equation for peak area and acetate concentration was y = 3.0069x + 0.7708 (R^2^ = 0.9776). x was peak area and y was acetate concentration.


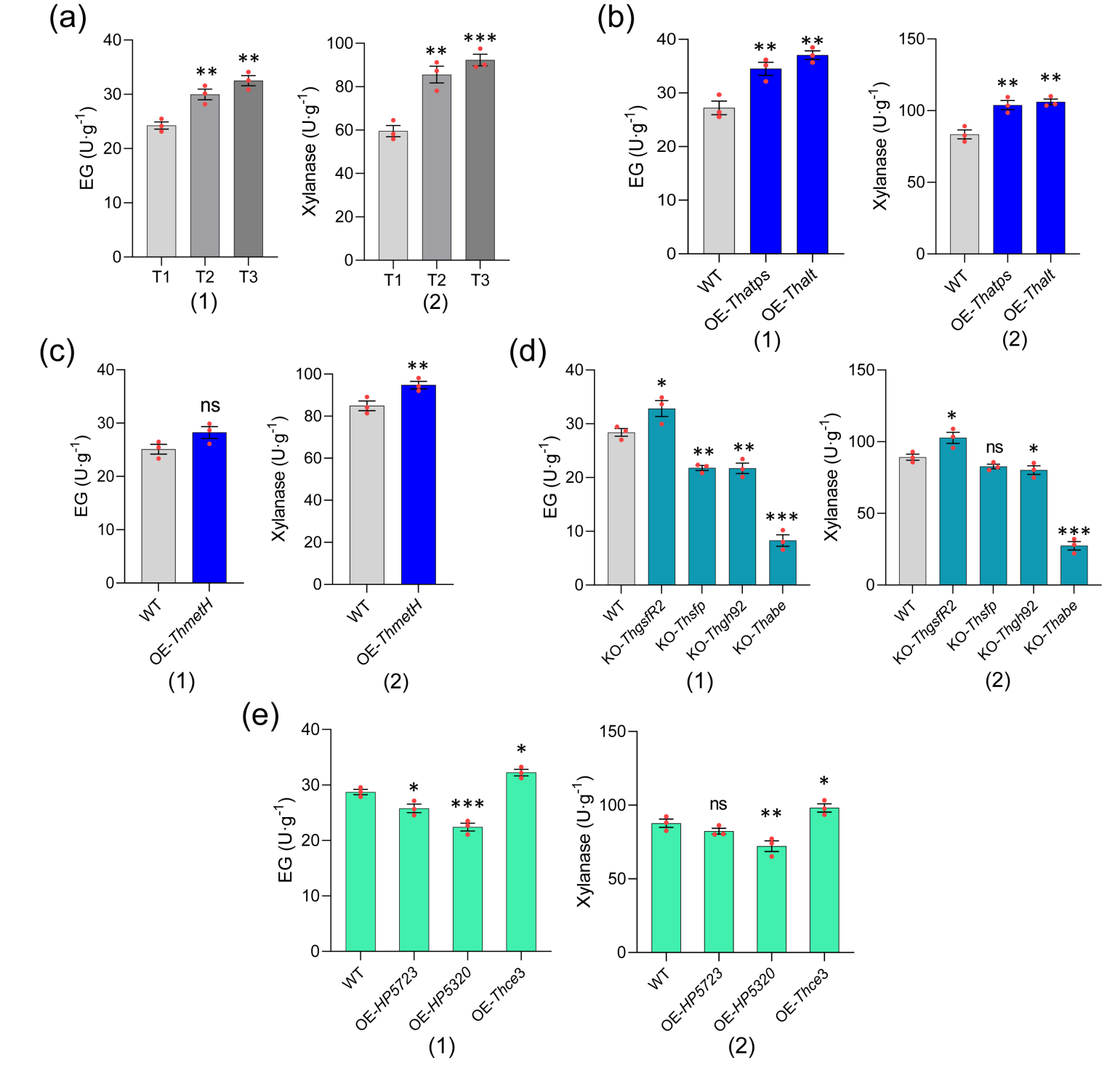


**Fig. S6 Endoglucanase (EG) activities and xylanase activities of treatments and mutants. (a)** EG and xylanase activities of AS gradients (T1, T2, and T3). **(b)** EG and xylanase activities of WT, OE-*Thatps*, and OE-*Thalt*. **(c)** EG and xylanase activities of WT, OE-*ThmetH*. **(d)** EG and xylanase activities of WT, KO-*ThgsfR2*, KO-*Thsfp*, KO-*Thgh*92, and KO-*Thabe*. **(e)** EG and xylanase activities of WT, OE-*HP5723*, OE-*HP5320*, OE-*Thce*3. Bars represent mean ± SEM, with n = 3 biological repeats; red dots resemble values from individual experiments. Student’s *t*-testing was conducted in (a, b, c, d, e). *signiﬁcant difference to WT at two-tailed *P* = 0.03 (d1, KO-*ThgsfR*2), 0.017 (d2, KO-*ThgsfR*2), 0.047 (d2, KO-*Thgh*92), 0.02 (e1, OE-*HP5723*) , 0.014 (e1, OE-*Thce*3), 0.033 (e2, OE-*Thce*3). **signiﬁcant difference to WT at two-tailed *P* = 0.159 (a1, T2), 0.001 (a1, T3) , 0.001 (a2, T2), (b1, OE-*Thatps*), 0.001 (b1, OE-*Thalt*) , 0.002 (b2, OE-*Thatps*) , 0.001 (b2, OE-*Thalt*), 0.003 (c2, KO-*ThmetH*), 0.001 (d1, KO-*Thsfp*), 0.001 (d1, KO-*Thgh*92), 0.005 (e2, OE-*HP5320*). ***signiﬁcant difference to WT at two-tailed *P* = 0.000 (a2, T3), 0.000 (d1, KO-*Thabe*), 0.000 (d2, KO-*Thabe*), 0.000 (e1, OE-*HP5320*). ns = no statistical difference to WT at two-tailed *P* = 0.207 (c1, KO-*ThmetH*), 0.131 (d2, KO-*Thsfp*), 0.219 (e2, KO-*ThHP5723*).
